# Polymer Binder Blends Stabilize Alkaline Hydrogen Evolution by Heterogenized Molecular Phen-Based Cobalt Electrocatalysts through Coordination and Environmental Control

**DOI:** 10.1021/jacs.4c18295

**Published:** 2025-03-11

**Authors:** Elizabeth K. Johnson, Daniel P. Musikanth, Christopher K. Webber, T. Brent Gunnoe, Sen Zhang, Charles W. Machan

**Affiliations:** Department of Chemistry, University of Virginia, PO Box 400319, Charlottesville, Virginia 22904-4319, United States

## Abstract

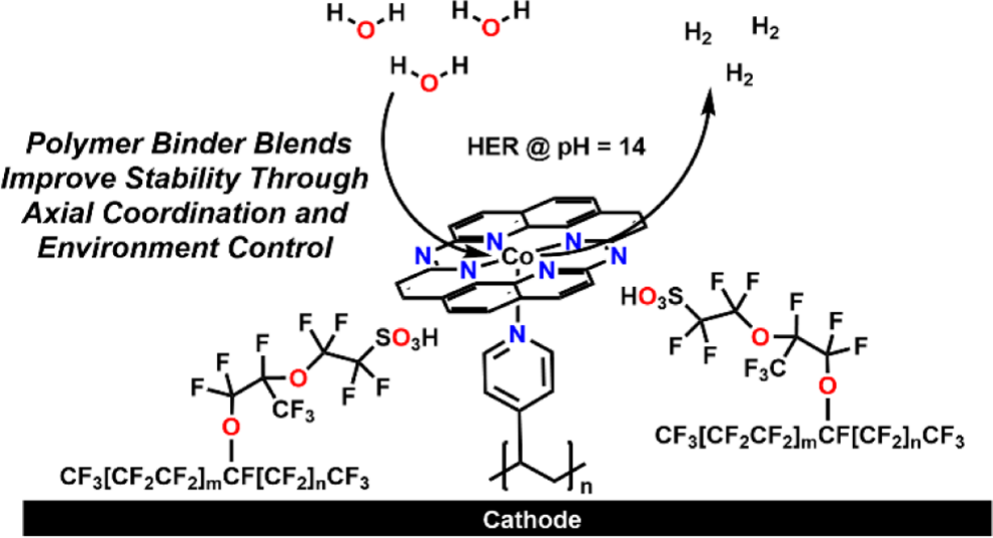

With the increase in greenhouse gas emissions and their detrimental effect on the environment, there is a push to develop a renewable way to produce H_2_, a fuel source that has nonharmful byproducts, unlike traditional methods of energy production. Alkaline water electrolysis has seen increasing focus as a viable way to produce H_2_, but efficient and stable electrocatalysts are required to facilitate this process. Here, a heterogenized Co(II) phenanthroline-based complex for the production of H_2_ from alkaline water is disclosed. Activity was improved by considering the role of axial Co ligation and the reaction environment created by the polymer binder in the catalyst inks by using variable ratios of Nafion and poly-4-vinylpyridine (P4VP). A ratio of 1:1 Nafion:P4VP was found to have the highest stability, and Co nanoparticle formation was not observed when P4VP was included as part of the binder mixture. The activity and stability enhancement could not be replicated by the addition of molecular pyridine or the use of poly-2-vinylpyridine, which is sterically prevented from coordinating to Co. The increased electrochemical performance caused by the inclusion of Nafion as part of the polymer binder is attributed to a role in mass transfer to and from the Co active site during catalysis, complementing the stabilizing effect of P4VP on the molecular active site.

## Introduction

Since the Industrial Revolution, the use of petrochemicals as fuels and as feedstocks for deriving basic commodity chemicals has risen exponentially.^1^ Because much of this utilization is based around combustion, there has been an accompanying increase in anthropogenic CO_2_ emissions, leading to atmospheric accumulation. Elevated concentrations of CO_2_ and other greenhouse gases are detrimental to the environment, leading to ocean acidification, bleaching of coral reefs, the melting of polar ice caps, and an increase in the frequency and severity of wildfires, droughts, and other extreme weather patterns.^1^ Carbon-free fuel sources are an attractive alternative for energy generation in efforts to mitigate climate change. One of the most promising clean fuels is H_2_, as the only byproduct of H_2_ combustion or electrochemical oxidation during energy production is water.^2^ However, H_2_ is currently predominantly produced in a nonrenewable way, through steam reforming.^3^ Thus, there is an urgent need for a renewable production method of H_2_ that can be done at the scale needed to meet the world’s energy demands.

The electrochemical splitting of water in electrolysis cells can be used to produce green H_2_, if the electricity comes from a renewable energy source, such as solar or wind. The primary technologies of interest generally fall into three categories. First, there is a polymer electrolyte membrane electrolysis (PEMEL) cell, which operates with an acidic electrolyte.^4^ Acidic operating conditions require the use of scarce and expensive catalysts based on metals such as Ir and Pt.^4^ The second type of cell is an alkaline electrolysis (AEL) cell, which uses extremely high concentrations of caustic electrolyte (e.g., 25–40 wt % KOH).^4^ Although these conditions favor the kinetically challenging oxygen evolution reaction (OER), they are corrosive to the cheaper and more abundant Ni, Co, and Fe catalysts that are commonly employed.^4^ The third type of cell is the hydroxide exchange membrane electrolysis (HEMEL) cell, which can operate with much more dilute concentrations of a basic electrolyte (e.g., 1 M KOH) or in neutral water.^4^ These conditions can enable the use of less expensive transition metal catalysts for the OER and the hydrogen evolution reaction (HER); however, current cell designs and catalyst materials are still behind the PEMEL options in terms of performance. The potential savings in catalyst materials continue to garner interest in the development of new HEMEL cell designs for renewable H_2_ fuel production and how these might be made feasible at the industrial scale.

Primarily, nanomaterials are studied as catalysts for the HER under alkaline conditions. However, the use of molecular transition metal complexes has many potential benefits.^5−10^ Molecular transition metal complexes have a well-defined coordination site, often allowing for a much deeper understanding of the catalytic active site when compared to nanostructured materials that may contain a variety of potential active sites.^11^ Additionally, synthetic tuning of a molecular catalyst can be done to improve catalysis through mechanistic testing and iterative design strategies. For testing scalability and in-device architectures based on aqueous solvent systems, molecular catalysts can be immobilized using conductive carbon materials.^12^

There have been many reports of homogeneous systems for the HER based on porphyrinic complexes like Co corrole.^13−15^ The Gross group has explored a range of Co(III) complexes in homogeneous conditions, finding that when the ligand backbone is substituted with fluorine atoms, the reduction potential shifts to less negative values in acidic conditions.^13^ Other Co(III) systems have been tested in homogeneous acidic conditions, some reaching TOFs of 600 s^–1^ and overpotentials as low as 247 mV.^15^ Bifunctional Co(III) systems have also been published, showing high activity for both the HER and the OER.^16−19^ When heterogenized onto a glassy carbon electrode, Co complexes have even demonstrated high activity for the OER in alkaline conditions and good activity for the HER in acidic conditions.^14^ Our group has also published a bifunctional Co(III) system based on perfluorinated corrole ligand supports modified with amines, showing high activity for both the HER and the OER in alkaline conditions.^20^

**Figure 1:**
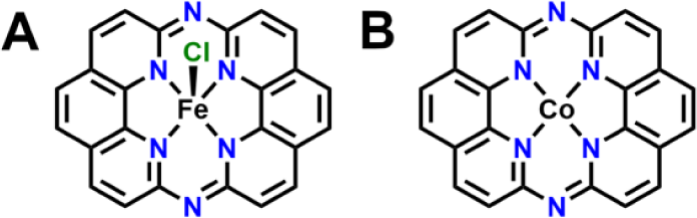
(A) Fe(phen_2_N_2_)Cl catalyst studied for ORR by Surendranath group in 2020.^11^ (B) Co(phen_2_N_2_) complex studied in this paper.

In searching for alternative platforms which could interface well with carbon materials, it was reasoned that molecular complexes with macrocyclic phenanthroline-based ligand frameworks could serve as effective catalysts for the HER under alkaline conditions due to their high rigidity and aromaticity.^21^ The high rigidity of the macrocyclic and dianionic pyridinic binding motif should act to stabilize the active site during redox changes, while the planar aromatic character should enable effective interfacing with electrode materials in a heterogeneous configuration. Indeed, a phenanthroline-based Fe(phen_2_N_2_)Cl complex (Figure 1) was shown by the Surendrenath group to be an effective catalyst for the oxygen reduction reaction (ORR) under acidic conditions (pH = 1).^11^ Here, the use of the cobalt analogue Co(phen_2_N_2_) complex for alkaline HER (pH = 14) is reported.

**Figure 2:**
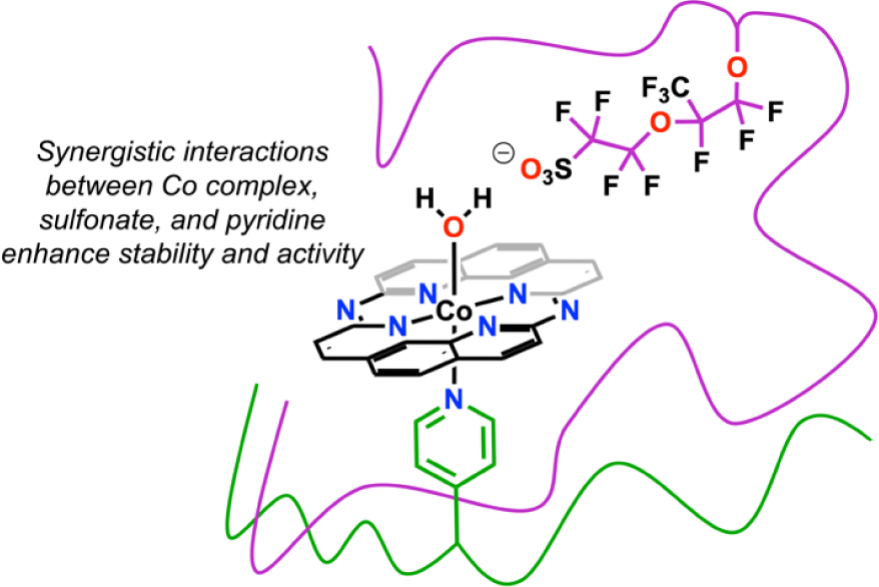
Mechanistic studies suggest that synergistic effects between Co complex, sulfonate, and pyridine achieve activity not intrinsic to the individual components.

Previous studies by McCrory and coworkers have shown that for the CO_2_ reduction reaction, when a pyridine-based polymer, poly-4-vinylpyridine (P4VP), is used to immobilize Co-based phthalocyanine electrocatalysts structurally related to the Co(phen_2_N_2_) complex, both the Faradaic efficiency for carbon-containing products and TOF were increased due to three main reasons: the Co metal center prefers axial ligand binding during catalysis, H-bonding interactions help stabilize intermediates, and proton relay effects can occur through the pyridyl groups.^22^ Based on this, it was also reasoned that there would be similar benefits to heterogenizing the Co(phen_2_N_2_) complex with this polymer to increase the nucleophilicity of the Co center for HER and introduce secondary reaction environment effects.^23^ Here, studies on the use of different ratios of Nafion and P4VP as a catalyst binder for the alkaline HER (pH = 14) reveal that axial ligand binding enhances activity at lower potentials and inhibits Co nanoparticle formation. Notably, the combination of Nafion and P4VP is required for the best catalytic performance, demonstrating both a more positive onset potential and less voltage change during chronopotentiometric stability tests. This suggests that water and proton transport is inhibited in P4VP only at elevated pH values.^24^ Further, mechanistic studies imply that there is a synergistic effect obtained in combining Nafion and P4VP that produces catalytic properties not available for either binder component individually (Figure 2).

## Experimental Section

### Synthesis and Characterization

The ligand precursor ((phen_2_N_2_)H_2_)^11^ and the Co(phen_2_N_2_) complex were synthesized as previously reported.^25^ Purified products were characterized by NMR (Figure S1), UV–vis (Figures S2 and S3), and elemental analysis. Characterization of [Co] following electrochemical testing was performed by transmission electron microscopy (TEM; Figures S50–S54), scanning electron microscopy-energy- dispersive X-ray spectroscopy (SEM-EDS; Figures S24–S49), and inductively coupled plasma atomic emission spectroscopy (ICP-OES). All TEM images were obtained on an FEI Tecnai Spirit (120 kV). All SEM-EDS images were obtained using an FEI Quanta 650 scanning electron microscope with EDS. ICP samples were prepared by heating a prepared electrode in 1.5 mL of nitric acid at 80° C for 30 min. The sample is then diluted to 10 mL with DI water and syringe filtered. All electrochemical experiments were completed using either a BioLogic VSP bipotentiostat equipped with a 20A/20 V booster or a BioLogic SP150E bipotentiostat equipped with a 20A/20 V booster.

### Catalyst Ink Preparation and Electrochemistry

To prepare the catalyst ink solution for all electrodes, 1 mM Co(phen_2_N_2_) was suspended with Vulcan Carbon (12.3 mg) in a solution of IPA (0.8 mL), DCM (2.6 mL), and EtOH (1.0 mL), along with a solution of the desired polymer binder (0.5 mL). Polymer binders (P4VP, Nafion, P2VP, Sustanion) were prepared as stock solutions of 5 wt %, and when more than one polymer was used in an ink formulation, the total volume of the blend added to the catalyst ink remained 0.5 mL. For 1:3 Naf:P4VP, 0.125 mL of Nafion and 0.375 mL of P4VP stock solutions were combined; for 1:1 Naf:P4VP, 0.25 mL of Nafion and 0.25 mL of P4VP; and for 3:1 Naf:P4VP, 0.375 mL of Nafion and 0.125 mL of P4VP. After sonication of the catalyst ink, 400 μL of the ink solution were dropcast onto a 1 cm^2^ area of EP40 carbon paper using a micropipettor. The electrode was dried in ambient conditions for 30 min, and linear sweep voltammetry in the catalytic regime was taken until the response overlaid on consecutive scans before use in further testing (ca. 2 to 6 scans).

Onset potential was determined by plotting the most linear portion of the LSV data (−5 mA cm^2^ to −100 mA cm^2^, generally) and applying a linear fit to obtain the x-intercept. Overpotential was determined by calculating the normalized difference between the thermodynamic potential of the HER in alkaline conditions (0 V vs RHE)^26^ and the onset potential calculated using the method listed above. To determine Tafel slopes, LSVs were taken in consecutive scans until the response was overlaid, as described above. This is to ensure that any material that is weakly adsorbed is removed before the Tafel studies are conducted. Then, chronoamperometry experiments were conducted for 3 min starting at the potential where −10 mA/cm^2^ was reached, going to more negative potentials in increments of 20 mV until 10–12 chronoamperometry experiments were completed. The log of the final current density of the chronoamperometry was plotted against potential vs RHE to obtain the Tafel slope of the system.

## Results

### HER Studies

**Figure 3:**
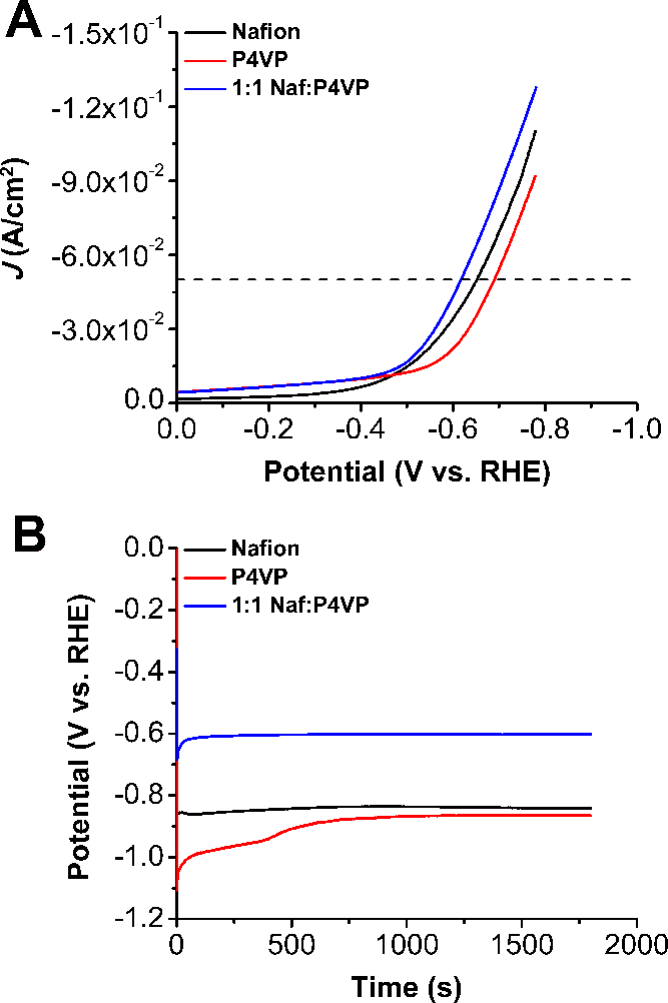
(A) Comparison of LSVs of [Co] with Nafion, P4VP, and 1:1 Naf:P4VP. (B) Chronopotentiometry experiments of [Co] with Nafion, P4VP, and 1:1 Naf:P4VP at −50 mA/cm^2^.

**Figure 4:**
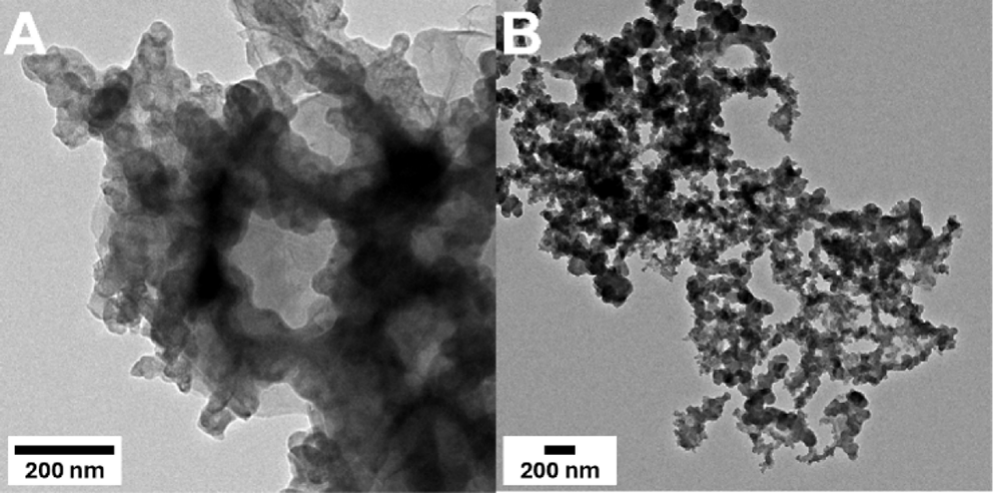
(A) TEM image of pure Nafion system and (B) pure P4VP system after 12 h chronopotentiometry experiment at −10 mA/cm^2^ current density. Nanoparticulate formation is not evident in either image.

All ratios of polymer binder used in the catalyst inks were tested for their heterogeneous HER performance at pH 14 (1 M KOH) by potentiostatic and galvanostatic techniques. When Nafion is exclusively used as the supporting polymer in the catalyst ink, linear sweep voltammograms (LSVs) were used to determine an onset potential of −0.52 V vs RHE, with an overpotential of 0.59 V at a benchmark current density of −50 mA/cm^2^ (Figures 3, S4, and Table S1). The Tafel response of catalytic systems can be used as a tool for mechanistic interrogation and for activity comparisons between different systems.^27^ The Tafel slope of the Nafion system is 265 mV/dec (Figure S6), significantly larger than the slope expected for the Volmer step of water dissociation, generally thought to be the likely RDS for alkaline HER.^28^ This deviation suggests that transport of water to the active site governs the observed catalytic response, suggesting that active sites are distributed within the polymer binder rather than exclusively exposed to the reaction solution. Chronopotentiometry experiments were subsequently performed to probe the catalyst longevity. The observed overpotential of this system when held at −10 mA/cm^2^ for 30 m begins at 0.54 V and decreases to 0.52 V, suggesting that the catalyst is relatively stable (Figure S4). However, when held at −10 mA/cm^2^ for 2 and 12 h, there is an appreciable change in cell voltage to more reducing potentials (Figure S5), suggesting that this system is not stable during extended time experiments. It should be noted that there were no Co nanoparticles found (Figure 4). This implies that although some change in the catalyst structure is occurring, it is not reconfiguring into a different species on the electrode.

**Table 1:** Change in Potential over 30 Min during Chronopotentiometry Experiments at −50 mA/cm^2^ with Nafion, P4VP, or 1:1 Naf:P4VP as the Polymer Binder

Polymer Binder	ΔPotential mV/s	Average V
Nafion	5.4(2) × 10^– 3^	–0.842(7) V
P4VP	4.3(3) × 10^– 3^	–0.896(45) V
1:1 Naf:P4VP	–1.7(2) × 10^– 2^	–0.604(5) V

Based on the hypothesis that coordinate covalent interactions between the polymer binder and the molecular catalyst could improve stability and activity, polymer poly-4-vinylpyridine (P4VP) was then used in place of Nafion. Inspired by the reports of McCrory and coworkers on CO_2_ reduction performance,^24,29,30^ this polymer was chosen to discourage aggregation of the Co complex (association of molecules through noncovalent interactions like pi-pi stacking) and inhibit Co nanoparticle formation from degradation (demetalation of the molecular species). With P4VP as the only polymer binder in the catalyst ink, the system had an onset potential of −0.57 V vs RHE, which is 50 mV more negative than the system with only Nafion polymer (Figure 3A and Table S1). Additionally, it reaches the −50 mA/cm^2^ benchmark at −0.67 V vs RHE, which is 80 mV more negative than the Nafion system. During the 30 m chronopotentiometry experiment at −50 mA/cm^2^, the Nafion system has an average voltage of −0.842 V, while that of P4VP is −0.896 V (Table 1 and Figure 3B).

The Tafel activity of the P4VP system has two slopes: 211 mV/dec at less negative potentials and an increase to 323 mV/dec as the applied potential becomes more negative (Figure S9). It is proposed that at more negative potentials, substrate depletion is sufficient with P4VP as the binder to require greater electrochemical driving force, owing to weak interactions between water and pyridine sites. As was the case for the experiments with Nafion as the exclusive binder, chronopotentiometry experiments with P4VP as the only polymer binder showed a decrease in stability over time, especially at more negative current densities (Figures S7 and S8). However, TEM images showed no nanoparticle formation (Figures 4 and S50) suggesting that similar to the Nafion system, P4VP experiences changes on the electrode surface at applied potential. Overall, the decreased performance then suggested that additional factors were inhibiting electrochemical performance, possibly related to substrate transport for HER at pH 14 in aqueous conditions and dissolution of the P4VP polymer and Co complex from the conductive support.

**Figure 5:**
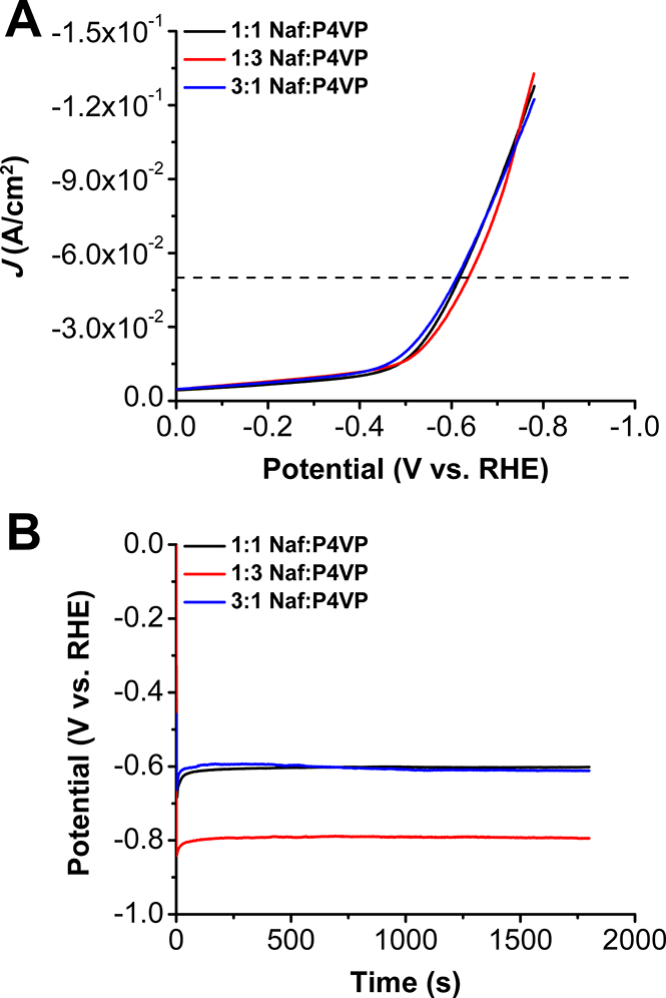
(A) Comparison of LSVs of [Co] with all Naf:P4VP conditions. (B) Chronopotentiometry at −50 mA/cm^2^ of [Co] with all Naf:P4VP conditions.

To test the possibility of improving the electrocatalytic performance, variable mixtures of both Nafion and P4VP polymers were tested as binding agents. Three different ratios of Nafion:P4VP were prepared (1:3, 1:1, and 3:1; note that the ratios refer to the relative composition of 5% by wt. polymer solution used in the ink formulation; see **Experimental**). Of these three systems, by LSV, the 3:1 ratio has the least negative onset potential at −0.52 V vs RHE (1:3 onset −0.55 V vs RHE; 1:1 ratio onset −0.52 V vs RHE) (Figure 5 and Table S1). The overpotential at −50 mA/cm^2^ follows a different trend than onset potentials, with the 1:1 ratio having the lowest overpotential at 0.63 V and the 3:1 ratio having the largest at 0.67 V. Of the three, the 1:3 Naf:P4VP blend showed the greatest instability in chronopotentiometry experiments (Figures S16proof and S17 and Table 2).

**Table 2:** Change in Potential over 30 Min of a −50 mA/cm^2^ Chronopotentiometry Experiment for All Polymer Blends of Nafion and P4VP

Conditions	ΔPotential mV/s	Average V
1:1 Naf:P4VP	1.2(7) × 10^–2^	–0.604(5) V
1:3 Naf:P4VP	1.8(7) × 10^–2^	–0.792(4) V
3:1 Naf:P4VP	3.7(3) × 10^–2^	–0.605(6) V

An evaluation of the Tafel slopes for all three mixed polymer inks (Figures S12, S15, and S18) showed similar slopes to that of the pure Nafion system (Figure S6), again implicating substrate transport to active sites in the catalyst polymer layer as rate-limiting: 3:1 was 219 mV/dec, 1:1 was 244 mV/dec, and 1:3 was 263 mV/dec. It should be acknowledged that this effect could also be reasonably attributed to H_2_ getting trapped within the polymer blends and therefore inhibiting activity, as described by others.^31−33^ The P4VP system showed the largest slope of 323 mV/dec at the most negative potentials assessed (Figure S9).

**Figure 6:**
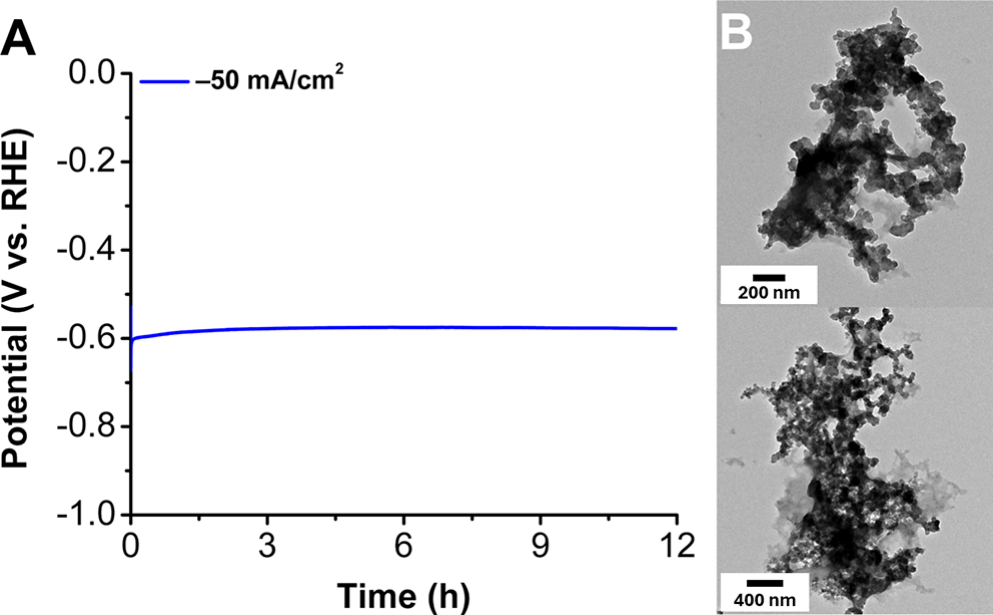
(A) Chronopotentiometry of [Co] with 1:1 Nafion:P4VP held at −50 mA/cm^2^ for 12 h. (B) TEM images of (A) after electrolysis. Average V = −0.605, standard deviation = 0.007.

Comparing chronopotentiometry and LSV experiments for all three blended systems revealed general stability relative to the pure Nafion or P4VP polymer binder systems (Figures S19 and S20). Overall, the 1:1 and 3:1 ratios are significantly more stable than all of the other systems tested, with the least amount of change in potential over time (Table 2) and the least negative onset potentials. During the 30 m chronopotentiometry experiments at −50 mA/cm^2^, the 1:1 and 3:1 binder ratios have average voltages of −0.604 and −0.605 V, respectively, which are significantly more positive than the pure Nafion and P4VP systems (Figures 3B, 5, Tables 1 and 2). The 1:3 ratio is much more negative than this, with an average voltage of −0.792 V, much closer to the values of the pure Nafion and P4VP systems. However, during longer experiments (2 and 12 h), the 3:1 ratio started to show significant voltage changes (Figure S17), while the 1:1 ratio remained much more stable (Figure S11). TEM images show no nanoparticle formation for any of these systems with blends of the two supporting polymers. Longer chronopotentiometry experiments were conducted for 12 h at −10 mA/cm^2^ for the conditions containing polymer blends, and no nanoparticles were observable by TEM (Figures S51–S53). Impressively, this stability was also observed for 12 h at −50 mA/cm^2^ for the 1:1 polymer binder composition; no nanoparticles were observed (Figures 6and S39), and a change in potential of 9.8(4) × 10^–^^4^ mV/s (3.1 mV/h) was measured.

**Figure 7:**
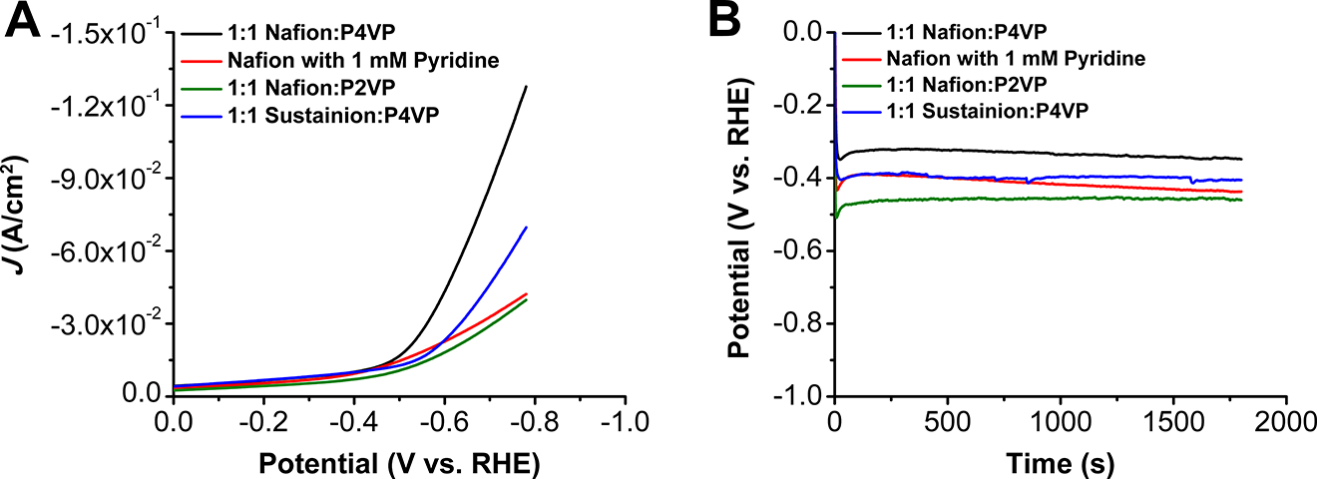
(A) Comparison of LSVs of [Co] with 1:1 Nafion:P4VP, Nafion with 1 mM pyridine, and 1:1 Nafion:P2VP. (B) Chronopotentiometry at −10 mA/cm^2^ of [Co] with 1:1 Nafion:P4VP, Nafion with 1 mM pyridine, and 1:1 Nafion:P2VP.

To evaluate whether the axial binding to P4VP was specifically necessary to improve stability and activity, three control experiments were conducted. First, an ink solution was made with pure Nafion as the polymer binder as before, but with 1 mM pyridine added. Second, a 1:1 ratio of Nafion:poly-2-vinylpyridine (P2VP) was added to the ink solution as the polymer binder. Finally, a 1:1 ratio of Sustainion:P4VP was added to the ink solution and tested. All of these experiments showed lower activity when compared to the 1:1 Nafion:P4VP system, and chronopotentiometry experiments showed that all controls had larger overpotentials at −10 mA/cm^2^ when compared to the 1:1 Nafion:P4VP system (Figure 7). While inclusion of pyridine in the catalyst ink could be expected to result in the formation of a five-coordinate species being deposited, since these are not attached to a high molecular weight polymer, they do not offer any advantage for stability (Figure S21). Likewise, the position of the heteroatom in P2VP should make axial coordination difficult due to steric reasons, since the N atom is now positioned *ortho* to the point of attachment to the polymer backbone (Figure S22), as described by McCrory and coworkers for cobalt phthalocyanine.^34^ Sustainion is an anion-exchange polymer, while Nafion is a cation-exchange polymer. It could be assumed that Sustainion would enhance activity since catalysis is occurring under alkaline conditions, where there are significant concentrations of hydroxide anions, but this was not shown (Figure S23).

**Figure 8:**
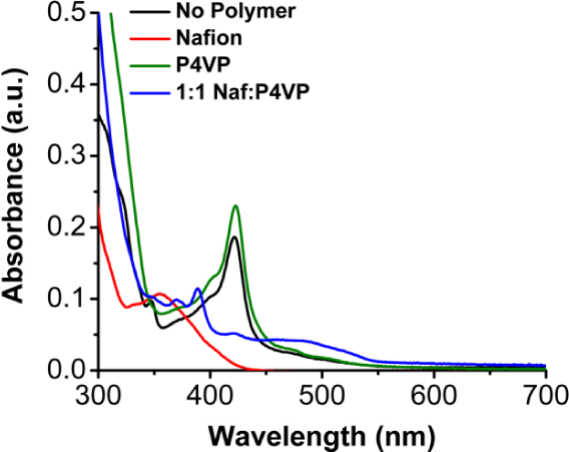
UV–vis spectra of 10 μM [Co] in 90% DMF and 10% polymer dissolved in a mixture of alcohols. The mixture of alcohols is composed of 39% 1-propanol, 39% IPA, 20% water, and 2% MeOH.

To more directly observe the environment of [Co] when different polymers are used, UV–vis experiments were performed. McCrory and coworkers have previously shown with cobalt phthalocyanines that absorbance corresponding to the molecular species increased when P4VP was introduced to the solution, which was attributed to better dispersion of the molecular Co species in solution.^29^ In order to more efficiently compare results across all conditions, P4VP was dissolved in a mixture of alcohols to mimic the commercial solution of Nafion. If aggregation of the complex was occurring (association through noncovalent forces like pi-pi stacking), the peak at 421 nm would blue-shift, and there would be a deviation from Beer’s law.^29^ For the Co(phen_2_N_2_) complex, a similar trend was observed, for which its primary absorbance in the visible region with λ_max_ = 421 nm showed an increase in intensity when P4VP was introduced at a [Co] concentration of 10 μM (Figures 8, S55 and S56). Interestingly, when Nafion is used as the binder, this absorption band at 421 nm disappears, and a new band appears at 354 nm, which could be attributed in part to aggregation of the cobalt complex, as well as the possibility of interactions between the sulfonates of the Nafion binder and the Co center (Figures 8 and S61). Interestingly, for the 1:1 combination of Nafion and P4VP, the absorption at 421 nm diminishes in intensity by about a third, and new bands appear at 480 nm, 388 nm, 370 nm, and 348 nm (Figures 8 and S63). This observation of new absorbance bands suggests that, while the dispersed structure of the Co complex obtained with pure P4VP (presumably with axial coordination of pyridine) is still present at 421 nm, new species are also forming with electronic environments that are different from either pure Nafion or pure P4VP environments. Given the significant improvement of electrocatalytic properties for the 1:1 polymer blend of Nafion and P4VP, it can be speculated that some of these new environments seen in the UV–vis data are of significant benefit to the observed activity, implying a synergistic effect for sulfonate and pyridine groups. Consistent with this, similar spectral changes to those observed with P4VP, Nafion, and their blends were not observed with added molecular pyridine or P2VP (Figures S54–S63).

## Discussion and Conclusions

A Co(II) phenanthroline-based molecular catalyst has been prepared and heterogenized for studies of the HER at pH = 14. The role of the polymer binder used was evaluated by using Nafion and P4VP in a variety of ratios. The pure Nafion system performed better electrochemically than pure P4VP; however, characterization by UV–vis suggests that axial ligand binding between the nitrogen on the pyridine in the polymer and the Co metal center helps to increase dispersion in the catalyst ink.^22−24,29,30^

To optimize the performance of this system, three different ratios of Nafion:P4VP were explored, in contrast to other approaches.^35^ Chronopotentiometric performance was enhanced in all cases relative to the pure polymer binders; however, the 1:1 ratio showed the best stability, performing at the lowest overpotential and having the lowest amount of potential change over a period of 12 h at −50 mA/cm^2^. The high stability and low overpotential of the 1:1 Nafion:P4VP system are noteworthy given that the nature of the molecular complex is consistent across catalyst formulations. As a result, the reduction potential of the catalytic active site should be relatively unchanged in these catalyst inks, and differences in performance can be ascribed in part to secondary sphere effects from the polymer blend. Interestingly, the 1:1 binder ratio showed the lowest turnover frequency per Co site of the blended binder formulations (Table S2), which could be related to the observed greater stability. To better understand the necessity of P4VP, three control experiments were conducted using ink solutions which compared Nafion with 1 mM pyridine to ink solutions containing either 1:1 Nafion:P2VP or 1:1 Sustainion:P4VP. All of these control systems showed lower electrochemical activity when compared to the 1:1 Nafion:P4VP and were less stable when held at a constant current over a period of time.

Solution-phase UV–vis experiments performed in the presence of a polymer binder showed that when P4VP was added to the solution, dispersion of the cobalt complex increased. Conversely, when Nafion was added to the solution, aggregation of the intact molecular complex occurred, leading to a dramatic change in the electronic environment of the Co complex. The blend of the two polymers retained some of the dispersed character but also suggested that the synergistic effect of the polymer binder was producing additional new Co environments, possibly by combining coordination interactions with the pyridine-containing P4VP polymer with the solution-phase environment favored by Nafion. Based on these data, it is proposed that strong axial ligand binding between the metal center and the polymer is necessary for even dispersion,^20^ but that subunits capable of favorable interactions with water and H^+^ enhance electrochemical activity, even at high pH. The implications of these conclusions are being tested in ongoing experiments but imply that the secondary environment of the electrocatalytic active site can be tuned without requiring the *de novo* development of new polymer supports.

## Supporting Information Available

Description of experimental details and methods, electrochemical data, UV–vis, and microscopy data (PDF)
