# Supplementary material for: Polymer Binder Blends Stabilize Alkaline Hydrogen Evolution by Heterogenized Molecular Phen-Based Cobalt Electrocatalysts through Coordination and Environmental Control
Source: J Am Chem Soc. 2025 Mar 11;147(12):10459–65. doi: 10.1021/jacs.4c18295 (PMC11951075; doi:10.1021/jacs.4c18295)
Supplement: Supplementary file 1 — ja4c18295_si_001.pdf [file ja4c18295_si_001.pdf]

Supporting Information For

Polymer Binder Blends Stabilize Alkaline Hydrogen Evolution by Heterogenized Molecular Phen-based Cobalt Electrocatalysts Through Coordination and Environment Control

Elizabeth K. Johnson, Daniel P. Musikanth, Christopher K. Webber, Sen Zhang, T. Brent Gunnoe, and Charles W. Machan*

*machan@virginia.edu; ORCID 0000-0002-5182-1138

ORCID: E.K.J. 0009-0003-1216-9110; D.P.M. 0009-0000-4603-8977; C.K.W. 0000-0003-4299-3541; S.Z. 0000-0002-1716-3741; T.B.G. 0000-0001-5714-3887

Department of Chemistry, University of Virginia,
PO Box 400319, Charlottesville, VA 22904-4319, USA

Materials and Methods

General

All chemicals and solvents (ACS or HPLC grade) were commercially available and used as received unless otherwise indicated. For all air-sensitive reactions HPLC-grade solvents were obtained as anhydrous and air-free from a PPT Glass Contour Solvent Purification System. The reactor used to synthesize the $(phen_2N_2)H_2$ ligand is Parr Instruments 2430HC2 vessel with a PTFE seal equipped with a 1000 psig Fike burst valve. UV-vis absorbance spectra were obtained on a Cary 60 from Agilent. A FEI Quanta 650 Field Emission Scanning Electron microscope was used to obtain the SEM-EDS data. An Everhart-Thornley detector (ETD) was used to collect secondary electrons (SE) – 15 kV is the accelerating voltage – with a spot size ranging from 4.0-5.5 depending on the sample. The magnification is 10,000x for all samples. Transmission electron microscopy (TEM) images, which provided insights into metal distribution before and after electrolysis, were obtained on a FEI Tecnai Spirit (120 kV). The mass content of Co on was quantified by using inductively coupled plasma optical emission spectrometry (ICP-OES) on a PerkinElmer Avio-200 ICP spectrometer. All reported current densities are based on the geometric area of the carbon paper electrode used.

Synthesis of $(phen_2N_2)H_2$

The $(phen_2N_2)H_2$ ligand was synthesized based on previous reports.[1] 2,9-dichloro-1,10,-phenanthroline (280 mg 1.10 mmol) was charged in a 25 mL cylindrical glass insert. The insert was then placed into a Parr Instruments pressure reactor equipped with a 1000 psig burst relief valve, and purged with nitrogen for 20 m. After the nitrogen purge, the reactor was pressurized with 85 psi anhydrous ammonia and sealed. The reactor was then heated to 300 ºC in a bed of aluminum beads using a heating mantle for 3 days, after which it was brought to room temperature and the residual ammonia was vented. The yellow/orange crude product was then suspended in MeOH, and TFA and acetic acid were added. The resulting suspension was heated to 70 ºC and stirred for 30 m at temperature. The solution was then filtered while hot, and the isolated solid was suspended in 4 M NaOH. The suspension was stirred overnight, filtered, and the solid was collected (77 mg, 21% yield). ^1H NMR (600 MHz, d-tfa) δ 8.87 (d, J = 8.9 Hz, 4H), 8.22 (s, 4H), 7.96 (d, J = 9.0 Hz, 4H). UV-Vis (DMSO): λ_{max} = 363 (9,850 M^{-1}•cm^{-1}). Anal. Calcd. for $(phen_2N_2)H_2$ + 0.33 NaOH: C, 72.11; H, 3.61; N, 21.02. Found: C, 72.26; H, 3.63; N, 21.12.

Synthesis of $Co(phen_2N_2)$

The $Co(phen_2N_2)$ catalyst was synthesized according to previously reported methods.[2] The $(phen_2N_2)H_2$ ligand (50 mg, 0.13 mmol), was weighed out and placed in a two-neck round-bottom flask fitted with a reflux condenser and magnetic stir bar. Nitrobenzene (7.0 mL, 68 mmol) was added and the solution was heated to reflux with stirring. While the solution was heating, Co(II) acetate tetrahydrate (0.10 g, 0.40 mmol) was dissolved in benzyl alcohol (6.0 mL, 58 mmol). Some gentle heating of the benzyl alcohol solution was

required for all of the metal salt to dissolve. Once the nitrobenzene reached reflux, the benzyl alcohol solution was pipetted in slowly. This solution was heated and stirred for 30 m, filtered using a fine glass frit, and collected (44 mg, 76% yield). The solid was washing with ether (30 mL) and DCM (30 mL), then placed in a vacuum oven overnight. UV-Vis (DMF): λ_{max} = 350 (14,200 $M^{-1}\bullet cm^{-1}$), 424 (25,800 $M^{-1}\bullet cm^{-1}$). Anal. Calcd. for Co($phen_2N_2$) + 0.33 H_2O: C, 64.15; H, 2.84; N, 18.70. Found: C, 63.86; H, 2.70; N, 18.46.

Ink Preparation

The ink was prepared by sonicating a solution of Co($phen_2N_2$) (1 mM) with Vulcan carbon (12.3 mg; XC-72) in a mixture of DCM (2.6 mL), IPA (0.8 mL), and EtOH (1.0 mL) along with a polymer binder stock solution (0.5 mL) or mixture of stock solutions (0.5 mL total volume). The polymer binder stock solutions were 5% Nafion in IPA by wt% or 5% P4VP in IPA by wt%. For the 1:3 Nafion:P4VP polymer binder blend, 0.12 mL 5% Nafion and 0.38 mL 5% P4VP stock solution were combined; for 1:1 Nafion:P4VP, 0.25 mL 5% Nafion and 0.5 mL 5% P4VP; for 3:1 Nafion:P4VP, 0.38 mL 5% Nafion and 0.12 mL 5% P4VP were added. After sonication, 400 µL of the ink solution was dropcast onto a 1 cm^2 area of EP40 carbon paper using an autopipettor. The electrode was dried in ambient conditions for 30 m prior to use.

Electrochemistry

All electrochemical experiments were completed using either a BioLogic VSP Bipotentiostat equipped with a 20A/20V Booster or a BioLogic SP150E Biopotentiostat equipped with a 20A/20V Booster. An aqueous mercury/mercury oxide (Hg/HgO) electrode was used as the reference electrode with 1 M KOH electrolyte. A glassy carbon rod was used as the counter electrode. Carbon paper coated with the catalyst ink (described above) held by a copper clamp and copper wire was the working electrode. All the studies were carried out in 1 M KOH that had been sparged with nitrogen. All electrochemical experiments were corrected for internal resistance. All voltammograms were obtained at scan rates of 10 mV/s. All potentials are referenced vs RHE.

Conversion of Hg/HgO Potential to RHE

A Hg/HgO electrode has been used as the reference electrode for studying the alkaline hydrogen evolution reaction (HER). The internal solution within the Hg/HgO electrode is 1 M KOH. All the potential values obtained with respect to Hg/HgO have been converted to RHE (reversible hydrogen electrode), using the Nernst Equation as described below.[3]

$$E_{RHE} = E_{Hg/HgO} + \left(2.303 \times \frac{RT}{F}\right) \times pH + E^{0}_{Hg/HgO}$$

Where $E_{Hg/HgO}$ is the potential measured with Hg/HgO as the reference electrode, R is the ideal gas constant (8.31432 $J\cdot K^{-1}\cdot mol^{-1}$), T is temperature (298.15 K), F is Faraday's constant (95485 $C\cdot mol^{-1}$), $E^{0}_{Hg/HgO}$ is the half-cell standard reduction potential of the Hg/HgO electrode (0.0983 V vs. SHE [standard hydrogen electrode] at 25º C).[4] For a 1 M KOH solution, pH is 14. Therefore, the equation reduces to:

$$E_{RHE} = E_{Hg/HgO} + 0.9271$$

Tafel Studies

LSV scans to remove weakly adsorbed material were obtained until a constant current-voltage response was obtained (2 to 7 scans, on average). The potential at which the catalytic current reaches –5 mA/cm^2 was selected as the initial point for the chronoamperometry studies. Beginning at this potential, chronoamperometry was recorded for 180 s, at which point the observed current had plateaued. Next, the potential was stepped at 20 mV intervals more negative and the process repeated to obtain 10 total measurements.

Preparation of ICP-OES Samples

A 1 cm^2 area of carbon paper was coated with 400 µL of the 1 mM ink solution described above using an automatic pipettor and allowed to air dry for 20 m. For a given catalyst ink, data were collected for 8 conditions: (1) no electrochemistry performed, (2) after obtaining LSV's until runs overlayed each other, (3) after chronoamperometry conditions at –10 mA/cm^2 for 30 m, (4) after chronoamperometry conditions at –20 mA/cm^2 for 30 m, (5) after chronoamperometry conditions at –30 mA/cm^2 for 30 m, (6) after chronoamperometry conditions at –50 mA/cm^2 for 30 m, (7) after chronoamperometry conditions at –10 mA/cm^2 for 2 h, and (8) after chronoamperometry conditions at –10 mA/cm^2 for 12 h. The electrode (carbon paper coated with ink) was heated in 1 mL concentrated HNO_3 for 20 m. The orange acid solution was then transferred into a graduated cylinder and diluted up to 10 mL with DI water. The solution was then filtered with a 25 mm diameter syringe filter, 0.45 µm PTFE membrane, prior to analysis.

Preparation of SEM-EDS Samples

A 1 cm^2 area of carbon paper was coated with 400 µL of 1 mM ink solution using an autopipettor and allowed to air dry for 20 m. For a given catalyst ink, data were collected for 8 conditions: (1) no electrochemistry performed, (2) after obtaining LSV's until runs overlayed each other, (3) after chronoamperometry conditions at –10 mA/cm^2 for 30 m, (4) after chronoamperometry conditions at –20 mA/cm^2 for 30 m, (5) after chronoamperometry conditions at –30 mA/cm^2 for 30 m, (6) after chronoamperometry conditions at –50 mA/cm^2 for 30 m, (7) after chronoamperometry conditions at –10 mA/cm^2 for 2 h, and (8) after chronoamperometry conditions at –10 mA/cm^2 for 12 h. The electrode (carbon paper with coated ink) was then sonicated in DI water to remove excess potassium from the surface before imaging.

Preparation of TEM Samples

A 1 cm^2 area of carbon paper was coated with 400 µL of 1mM ink solution using an autopipettor and allowed to dry for 20 m. For each set of catalysts, data were collected for 8 conditions: (1) no electrochemistry performed, (2) after obtaining LSV's until runs overlayed each other, (3) after chronoamperometry conditions at –10 mA/cm^2 for 30 m, (4) after chronoamperometry conditions at –20 mA/cm^2 for 30 m, (5) after chronoamperometry conditions at –30 mA/cm^2 for 30 m, (6) after chronoamperometry conditions at –50 mA/cm^2 for 30 m, (7) after chronoamperometry conditions at –10

mA/cm^2 for 2 h, and (8) after chronoamperometry conditions at –10 mA/cm^2 for 12 h. The electrode (carbon paper with coated ink) was then sonicated in isopropanol for 10 m. The IPA solution was then used for imaging.

Calculation of TOF

The turnover frequency (TOF) was calculated using the following equation:

$$TOF = \frac{I}{n \times F \times m}$$

Where *I* is the current, *F* is Faraday's constant, *m* is the moles of the catalyst (as calculated from ICP data), and *n* is the number of electrons transferred to generate one molecule of the product (n=2 for HER).[5]

Tafel Studies

First, LSV scans were obtained until a constant current-voltage response was obtained (2 to 7 scans, on average). The potential at which the catalytic current reaches –5 mA/cm^2 was selected as the initial point for the chronoamperometry studies. Beginning at this potential, chronoamperometry was recorded for 180 s, at which point the observed current had plateaued. Next, the potential was stepped at 20 mV intervals more negative, and the process was repeated to obtain 10 total measurements.

Synthesis and Characterization

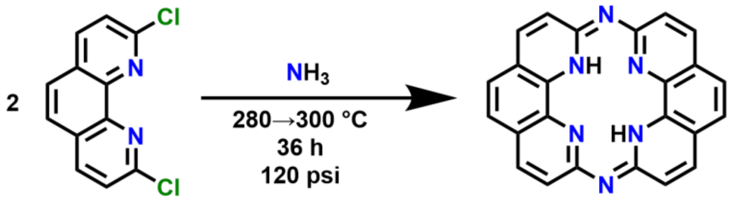

Scheme S1. Synthesis of $(phen_2N_2)H_2$ ligand

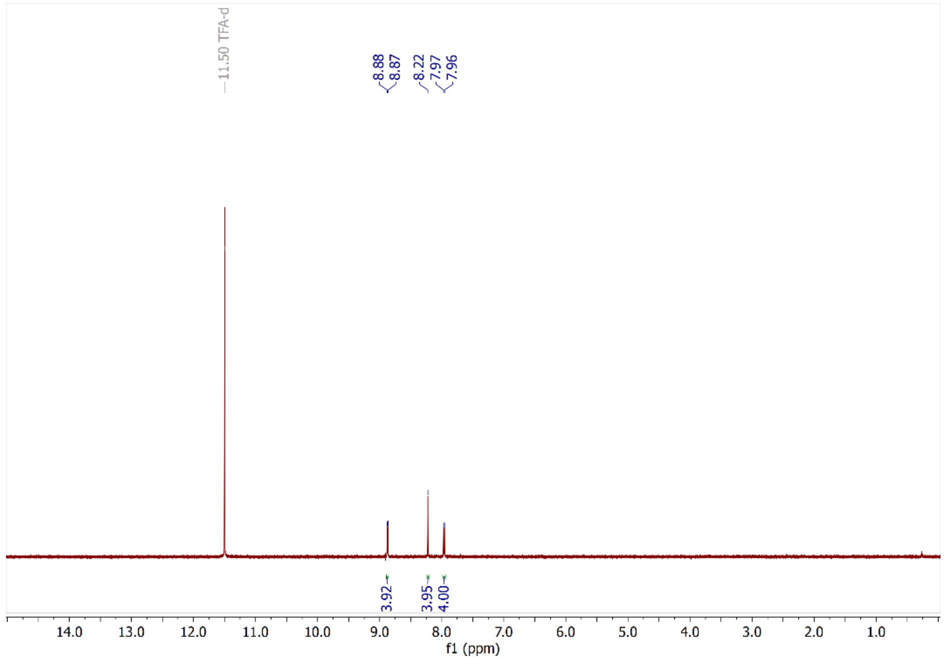

Figure S1. ^{1}H NMR of $(phen_2N_2)H_2$ ligand, recorded in *d*-TFA, 600 MHz.

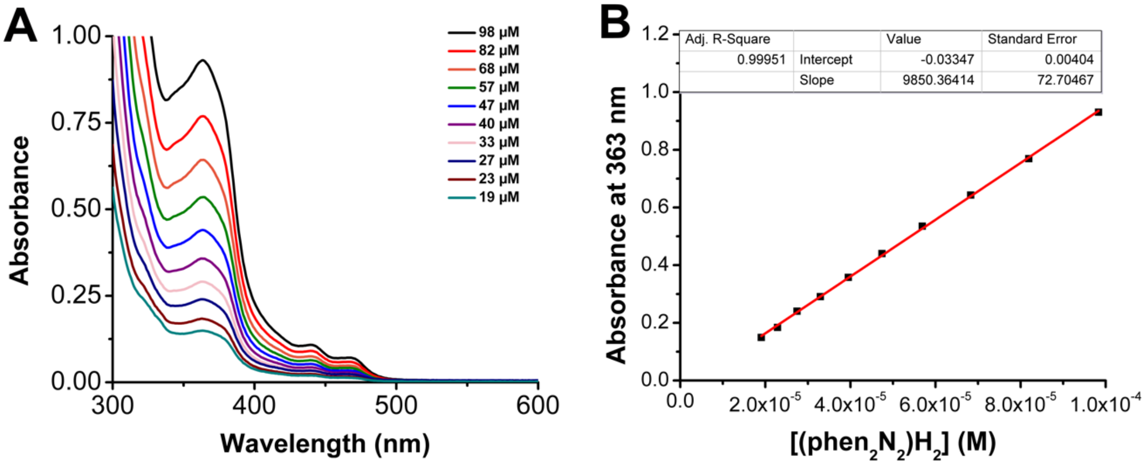

Figure S2. (A) UV-Vis spectrum of $(phen_2N_2)H_2$ ligand in DMSO. (B) Beer-Lambert plot of $(phen_2N_2)H_2$ ligand in DMSO at λ_{max} = 363 nm.

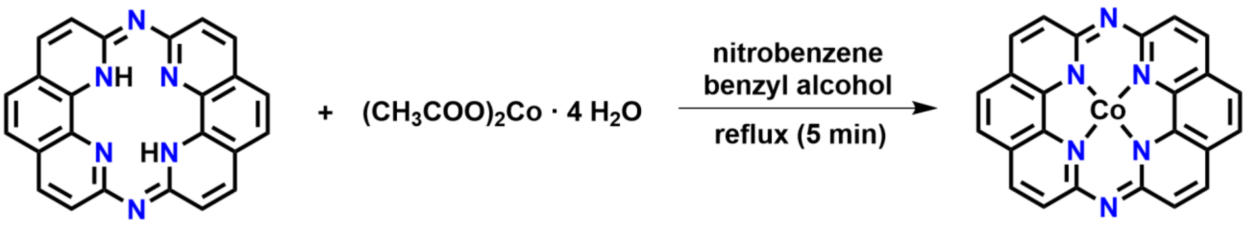

Scheme S2. Synthesis of $Co(phen_2N_2)$

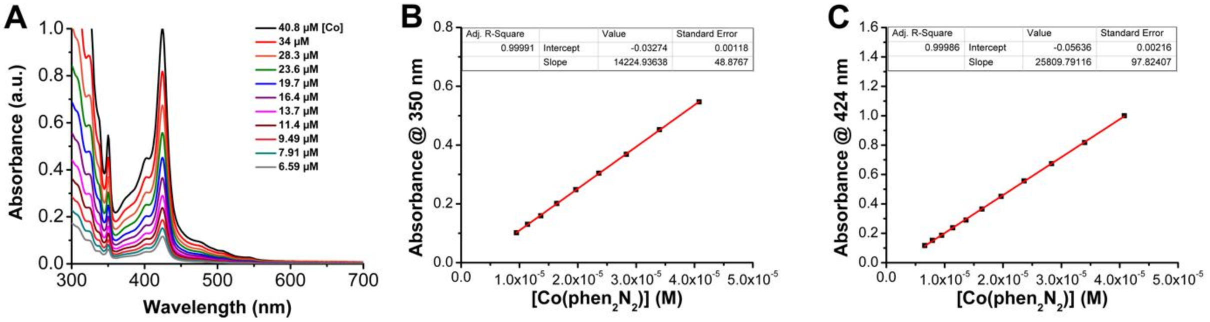

Figure S3. (A) UV-Vis spectrum of Co(phen$_2$N$_2$) in DMF. (B) Beer Lambert plot of Co(phen$_2$N$_2$) in DMF at λ_{max} = 350 nm. (C) Beer-Lambert plot of Co(phen$_2$N$_2$) in DMF at λ_{max} = 424 nm.

Electrochemistry

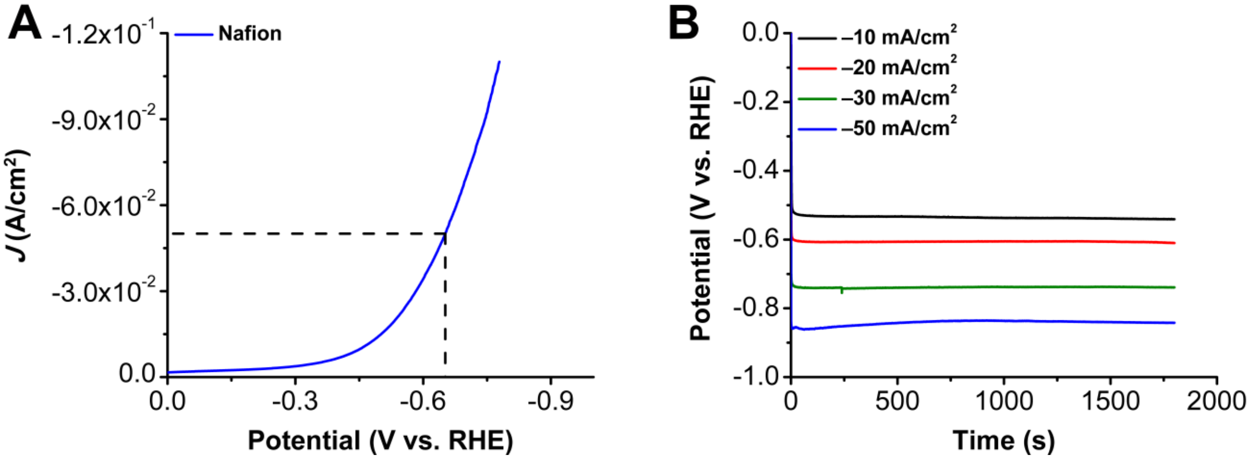

Figure S4. (A) LSV of 1 mM [Co]. (B) Stability tests of 1 mM [Co]. Conditions: ink solution containing 1 mM [Co], Vulcan Carbon, and Nafion. 1 cm^2 coated carbon paper with ink solution working electrode, glassy carbon counter electrode, Hg/HgO reference electrode. Performed under N$_2$ in 1 M KOH. 10 mV/s scan rate.

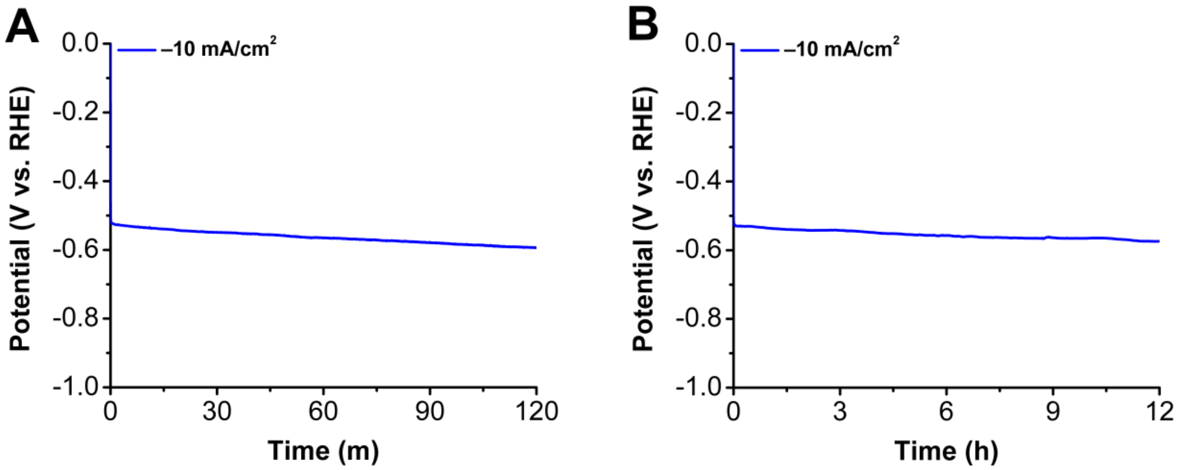

Figure S5. Stability tests of 1 mM [Co] of (A) 2 h and (B) 12 h. Conditions: Ink Solution containing 1mM [Co], Vulcan Carbon, and Nafion. 1 cm^2 coated carbon paper with ink solution working electrode, glassy carbon counter electrode, Hg/HgO reference electrode. Performed under N_2 in 1 M KOH.

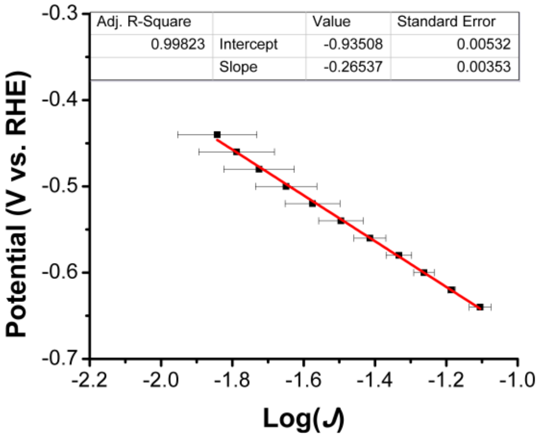

Figure S6. Tafel studes of 1 mM [Co]. Conditions: Ink Solution containing 1 mM [Co], Vulcan Carbon, and Nafion. 1 cm^2 coated carbon paper with ink solution working electrode, glassy carbon counter electrode, Hg/HgO reference electrode. Performed under N_2 in 1 M KOH.

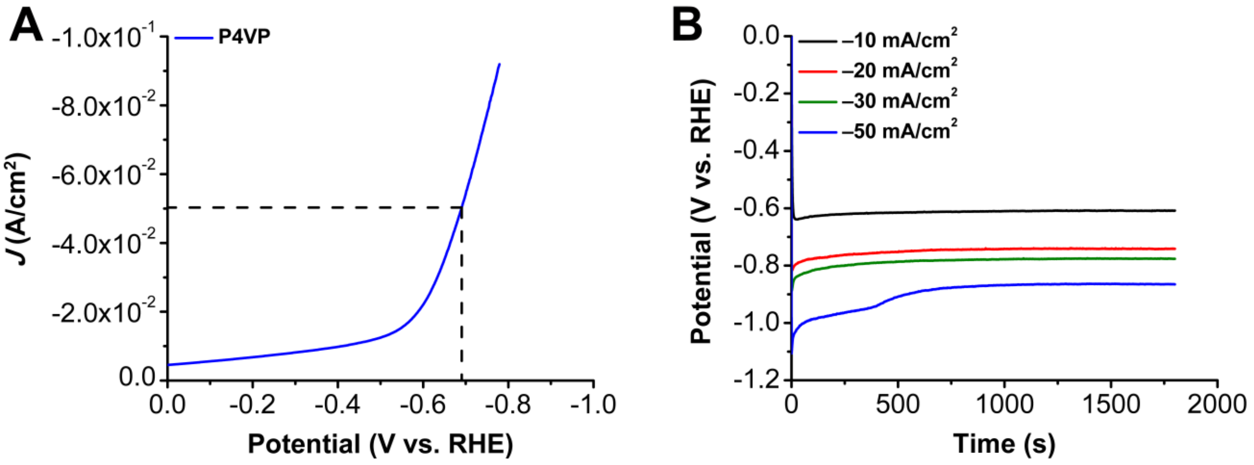

Figure S7. (A) LSV of 1 mM [Co] with P4VP. (B) Stability tests of 1 mM [Co] with P4VP. Conditions: Ink Solution containing 1 mM [Co], Vulcan Carbon, and P4VP 1 cm^2 coated carbon paper with ink solution working electrode, glassy carbon counter electrode, Hg/HgO reference electrode. Performed under N_2 in 1 M KOH. 10 mV/s scan rate.

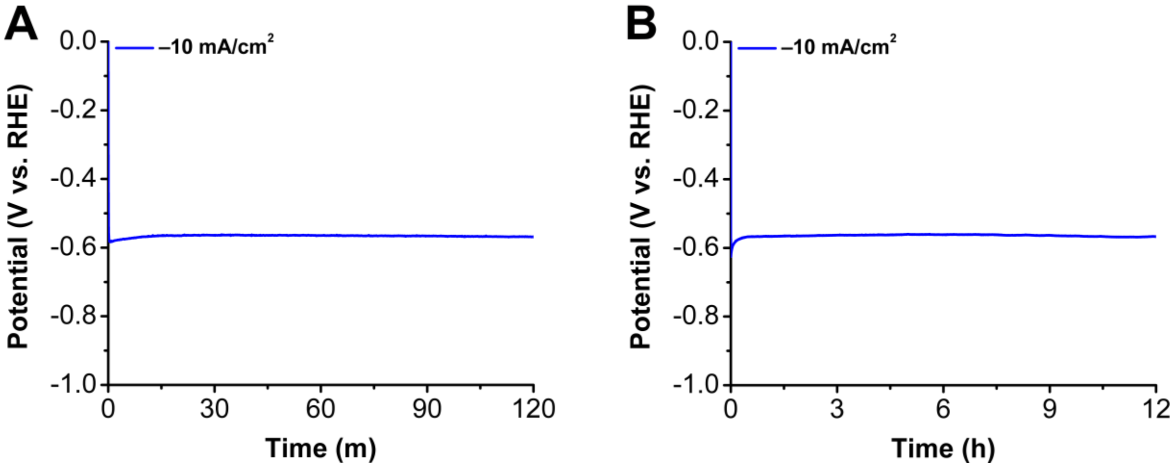

Figure S8. Stability tests of 1 mM [Co] of (A) 2 h and (B) 12 h. Conditions: Ink Solution containing 1mM [Co], Vulcan Carbon, and P4VP. 1 cm^2 coated carbon paper with ink solution working electrode, glassy carbon counter electrode, Hg/HgO reference electrode. Performed under N_2 in 1 M KOH.

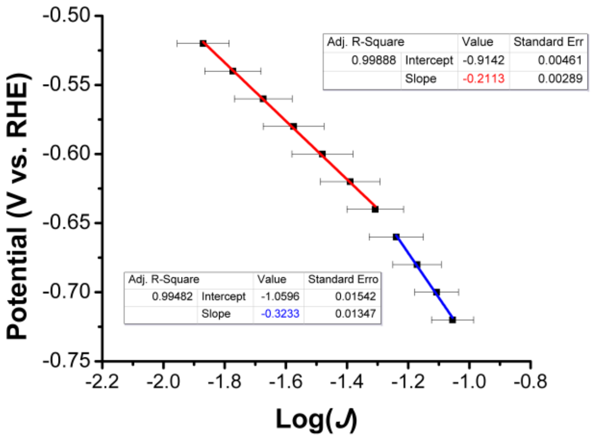

Figure S9. Tafel studies of 1 mM [Co] with P4VP. Conditions: Ink Solution containing 1mM [Co], Vulcan Carbon, and P4VP 1 cm^2 coated carbon paper with ink solution working electrode, glassy carbon counter electrode, Hg/HgO reference electrode. Performed under N_2 in 1 M KOH.

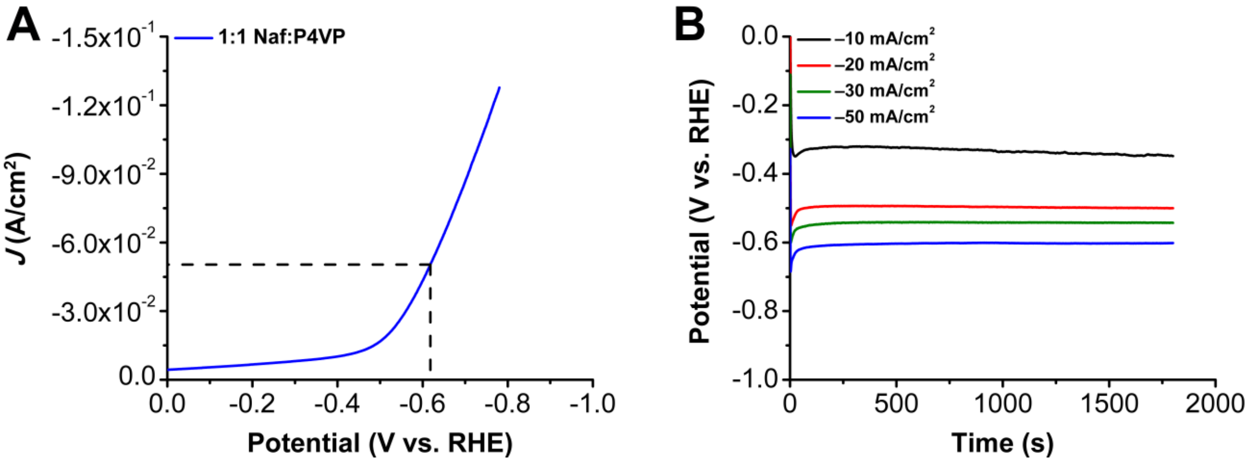

Figure S10. (A) LSV of 1 mM [Co] with 1:1 Naf:P4VP. (B) Stability tests of 1 mM [Co] with 1:1 Naf:P4VP. Conditions: Ink Solution containing 1mM [Co], Vulcan Carbon, and 1:1 Naf:P4VP 1 cm^2 coated carbon paper with ink solution working electrode, glassy carbon counter electrode, Hg/HgO reference electrode. Performed under N_2 in 1 M KOH. 10 mV/s scan rate.

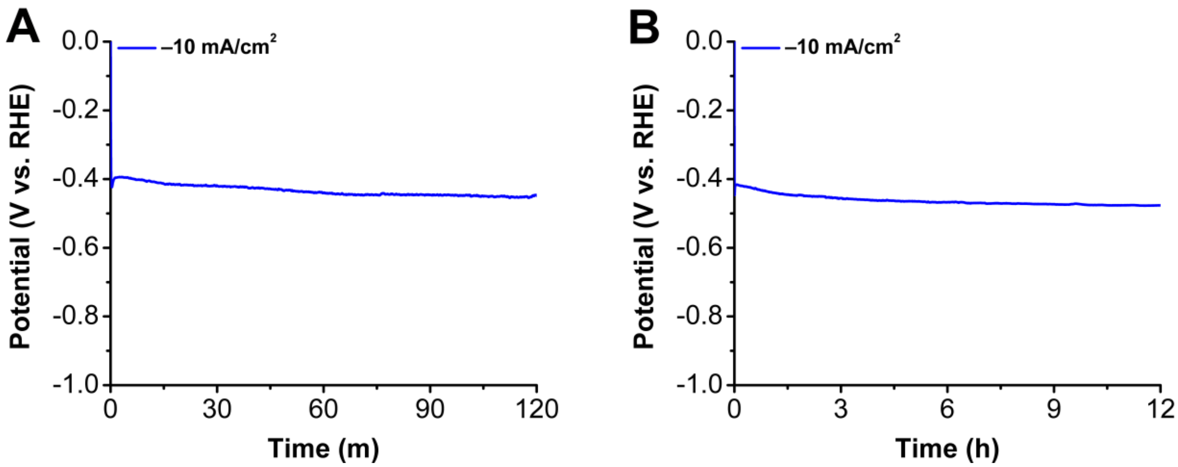

Figure S11. Stability tests of 1 mM [Co] of (A) 2 h and (B) 12 h. Conditions: Ink Solution containing 1 mM [Co], Vulcan Carbon, and 1:1 Naf:P4VP. 1 cm^2 coated carbon paper with ink solution working electrode, glassy carbon counter electrode, Hg/HgO reference electrode. Performed under N_2 in 1 M KOH.

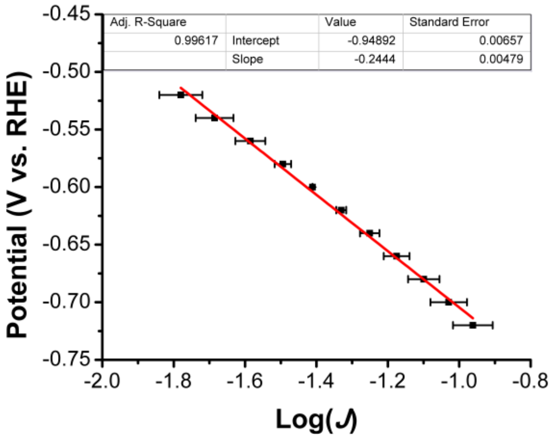

Figure S12. Tafel studies of 1 mM [Co] with 1:1 Naf:P4VP. Conditions: Ink Solution containing 1 mM [Co], Vulcan Carbon, and 1:1 Naf:P4VP 1 cm^2 coated carbon paper with ink solution working electrode, glassy carbon counter electrode, Hg/HgO reference electrode. Performed under N_2 in 1 M KOH.

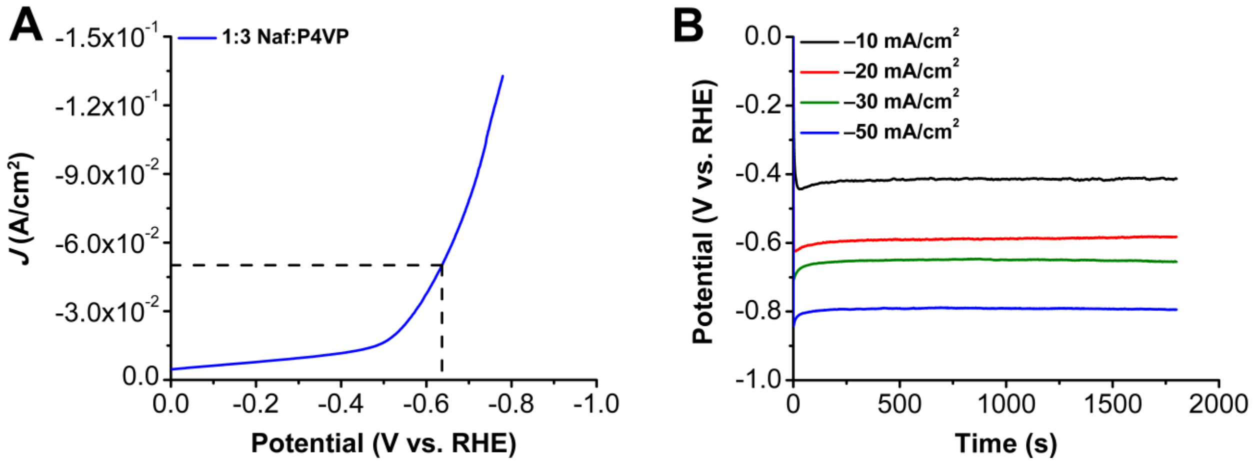

Figure S13. (A) LSV of 1 mM [Co] with 1:3 Naf:P4VP. (B) Stability tests of 1 mM [Co] with 1:3 Naf:P4VP. Conditions: Ink Solution containing 1mM [Co], Vulcan Carbon, and 1:3 Naf:P4VP 1 cm^2 coated carbon paper with ink solution working electrode, glassy carbon counter electrode, Hg/HgO reference electrode. Performed under N_2 in 1 M KOH. 10 mV/s scan rate.

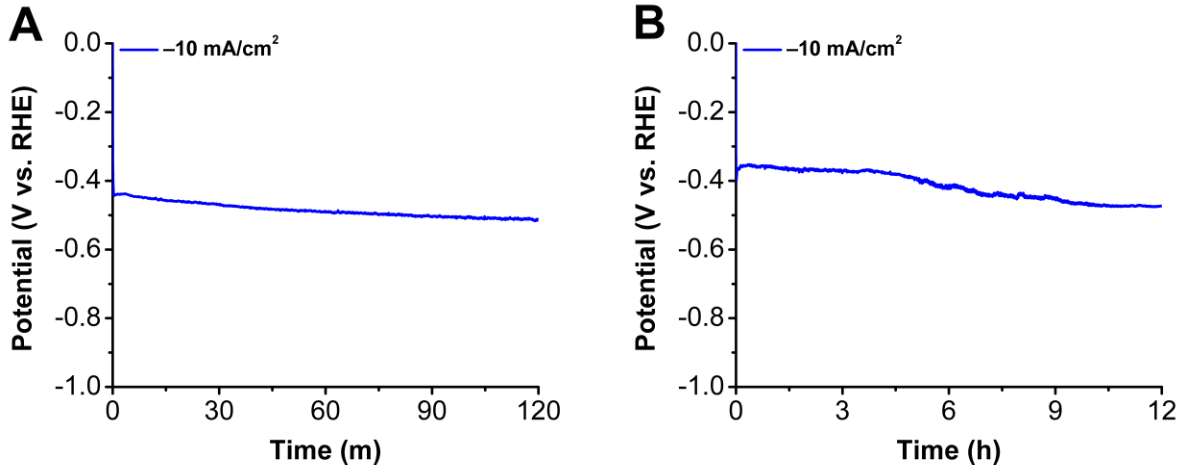

Figure S14. Stability tests of 1 mM [Co] of (A) 2 h and (B) 12 h. Conditions: Ink Solution containing 1mM [Co], Vulcan Carbon, and 1:3 Naf:P4VP. 1 cm^2 coated carbon paper with ink solution working electrode, glassy carbon counter electrode, Hg/HgO reference electrode. Performed under N_2 in 1 M KOH.

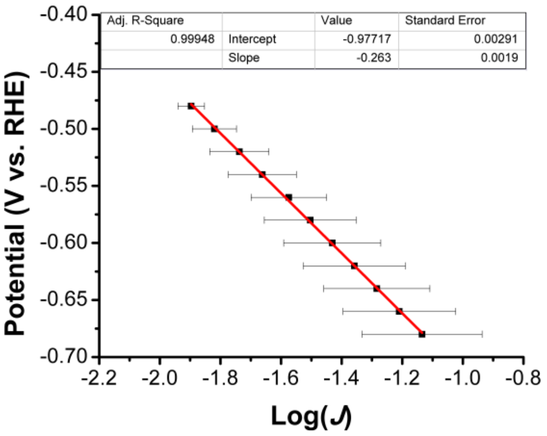

Figure S15. Tafel studies of 1 mM [Co] with 1:3 Naf:P4VP. Conditions: Ink Solution containing 1mM [Co], Vulcan Carbon, and 1:3 Naf:P4VP 1 cm^2 coated carbon paper with ink solution working electrode, glassy carbon counter electrode, Hg/HgO reference electrode. Performed under N_2 in 1 M KOH.

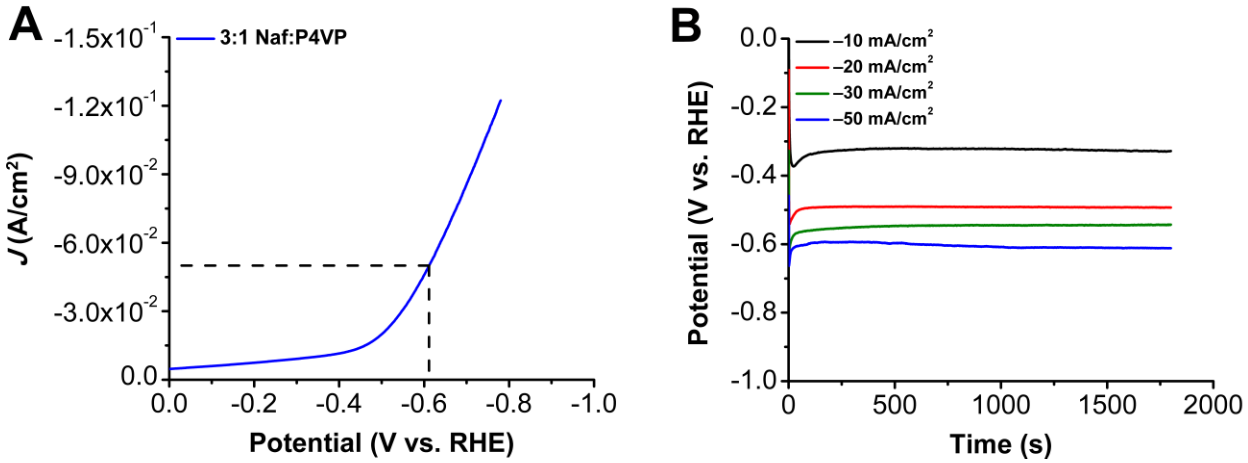

Figure S16. (A) LSV of 1 mM [Co] with 3:1 Naf:P4VP. (B) Stability tests of 1 mM [Co] with 3:1 Naf:P4VP. Conditions: Ink Solution containing 1mM [Co], Vulcan Carbon, and 3:1 Naf:P4VP 1 cm^2 coated carbon paper with ink solution working electrode, glassy carbon counter electrode, Hg/HgO reference electrode. Performed under N_2 in 1 M KOH. 10 mV/s scan rate.

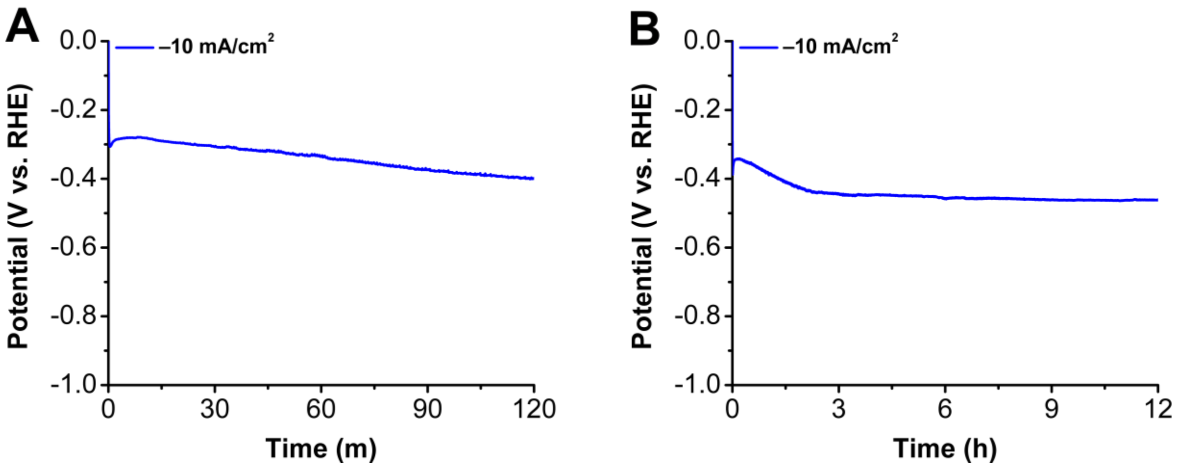

Figure S17. Stability tests of 1 mM [Co] of (A) 2 h and (B) 12 h. Conditions: Ink Solution containing 1mM [Co], Vulcan Carbon, and 3:1 Naf:P4VP. 1 cm^2 coated carbon paper with ink solution working electrode, glassy carbon counter electrode, Hg/HgO reference electrode. Performed under N_2 in 1 M KOH.

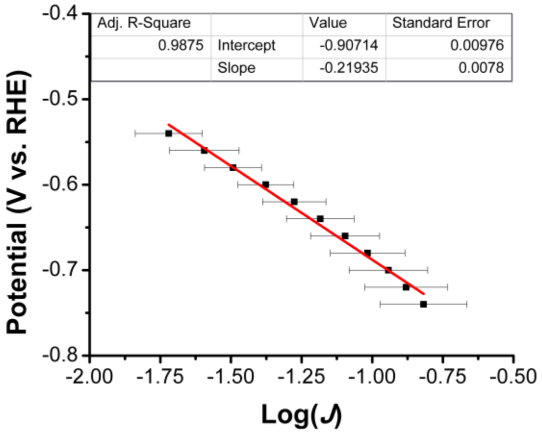

Figure S18. Tafel studies of 1 mM [Co] with 3:1 Naf:P4VP. Conditions: Ink Solution containing 1mM [Co], Vulcan Carbon, and 3:1 Naf:P4VP 1 cm^2 coated carbon paper with ink solution working electrode, glassy carbon counter electrode, Hg/HgO reference electrode. Performed under N_2 in 1 M KOH.

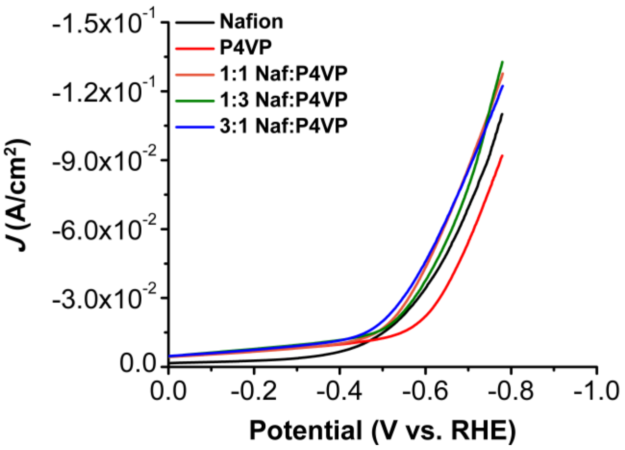

Figure S19. Overlay of LSVs of [Co] under all conditions.

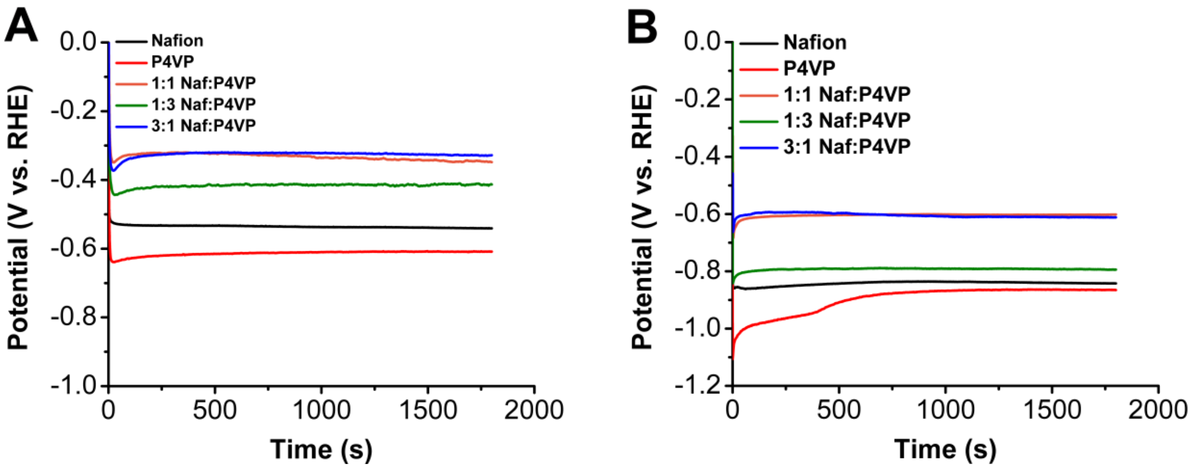

Figure S20. Chronopotentiometry experiments of all conditions at (A) –10 mA/cm^2 and (B) –50 mA/cm^2.

Table S1. Comparative summary of HER studies for all conditions.

	**Nafion**	**P4VP**	**1:1 Naf:P4VP**	**1:3 Naf:P4VP**	**3:1 Naf:P4VP**
**E_{onset} (V vs. RHE)**	–0.52(8)	–0.57(5)	–0.52(9)	–0.55(1)	–0.52(2)
**Reaches –50 mA/cm^2 (V vs. RHE)**	–0.59(4)	–0.67(3)	–0.63(1)	–0.66(4)	–0.67(5)
**Tafel Slope (mV/dec)**	265(3)	211(2), 323(1)	244(5)	263(2)	219(8)

Table S2. Calculation of TOF for all conditions using [Co] the values obtained from ICP-OES measurements.

		Nafion	P4VP	1:1 Naf:P4VP	1:3 Naf:P4VP	3:1 Naf:P4VP
**–10 mA/cm^2 (30 m)**	***I* (A)**	0.01	0.01	0.01	0.01	0.01
	n	2	2	2	2	2
	F	96485	96485	96485	96485	96485
	m	8.2(7) x 10^{-8}	1.4(7) x 10^{-8}	3.7(5) x 10^{-8}	1.2(5) x 10^{-8}	1.7(8) x 10^{-8}
	**TOF (s^{-1})**	**0.63(5)**	**3.7(2)**	**1.4(2)**	**4.4(2)**	**3.1(2)**
**–20 mA/cm^2 (30 m)**	***I* (A)**	0.02	0.02	0.02	0.02	0.02
	n	2	2	2	2	2
	F	96485	96485	96485	96485	96485
	m	8.2(7) x 10^{-8}	1.4(7) x 10^{-8}	3.7(5) x 10^{-8}	1.2(5) x 10^{-8}	1.7(8) x 10^{-8}
	**TOF (s^{-1})**	**1.3(1)**	**7.5(4)**	**2.9(4)**	**8.9(4)**	**6.2(3)**
**–30 mA/cm^2 (30 m)**	***I* (A)**	0.03	0.03	0.03	0.03	0.03
	n	2	2	2	2	2
	F	96485	96485	96485	96485	96485
	m	8.2(7) x 10^{-8}	1.4(7) x 10^{-8}	3.7(5) x 10^{-8}	1.2(5) x 10^{-8}	1.7(8) x 10^{-8}
	**TOF (s^{-1})**	**1.9(2)**	**11.2(6)**	**4.3(5)**	**13.3(5)**	**9.3(5)**
**–50 mA/cm^2 (30 m)**	***I* (A)**	0.05	0.05	0.05	0.05	0.05
	n	2	2	2	2	2
	F	96485	96485	96485	96485	96485
	m	8.2(7) x 10^{-8}	1.4(7) x 10^{-8}	3.7(5) x 10^{-8}	1.2(5) x 10^{-8}	1.7(8) x 10^{-8}
	**TOF (s^{-1})**	**3.1(3)**	**18.7(9)**	**7.2(9)**	**22.2(9)**	**15.5(8)**

Table S3. Calculation of change in potential for all conditions for chronopotentiometry experiments. Units are in mV/s.

	Nafion	P4VP	1:1 Naf:P4VP	1:3 Naf:P4VP	3:1 Naf:P4VP
**–10 mA/cm^2 (30 m)**	1.4(8) x 10^{-2}	–8.2(6) x 10^{-3}	–9.8(9) x 10^{-3}	–1.5(3) x 10^{-2}	–2.1(3) x 10^{-3}
**–20 mA/cm^2 (30 m)**	8.6(9) x 10^{-3}	–1.9(7) x 10^{-3}	–7.4(7) x 10^{-3}	–2.3(4) x 10^{-2}	–1.1(7) x 10^{-2}
**–30 mA/cm^2 (30 m)**	7.5(9) x 10^{-3}	–2.4(8) x 10^{-3}	–1.2(5) x 10^{-2}	–1.9(6) x 10^{-2}	–1.3(2) x 10^{-2}
**–50 mA/cm^2 (30 m)**	–5.4(2) x 10^{-3}	–4.3(3) x 10^{-2}	–1.2(7) x 10^{-2}	–1.8(7) x 10^{-2}	–3.7(4) x 10^{-2}
**–10 mA/cm^2 (12 h)**	1.6(3) x 10^{-3}	–8.5(6) x 10^{-4}	1.5(5) x 10^{-3}	1.3(1) x 10^{-3}	1.3(3) x 10^{-3}

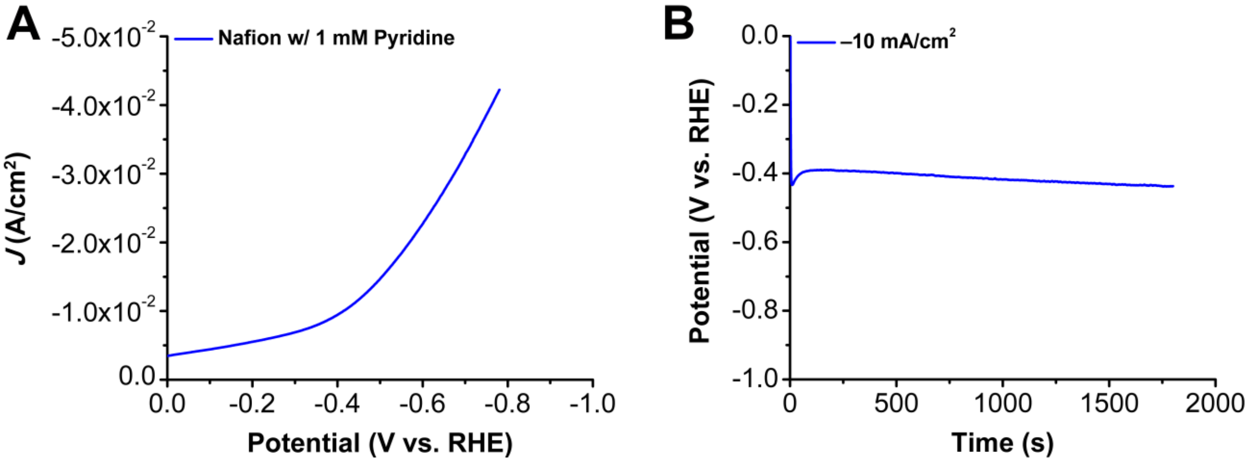

Figure S21. (A) LSV of 1 mM [Co] with 1 mM pyridine. (B) Stability test of 1 mM [Co] with 1 mM pyridine. Conditions: Ink Solution containing 1mM [Co], 1 mM pyridine, Vulcan Carbon, and Nafion. 1 cm^2 coated carbon paper with ink solution working electrode, glassy carbon counter electrode, Hg/HgO reference electrode. Performed under N_2 in 1 M KOH. 10 mV/s scan rate.

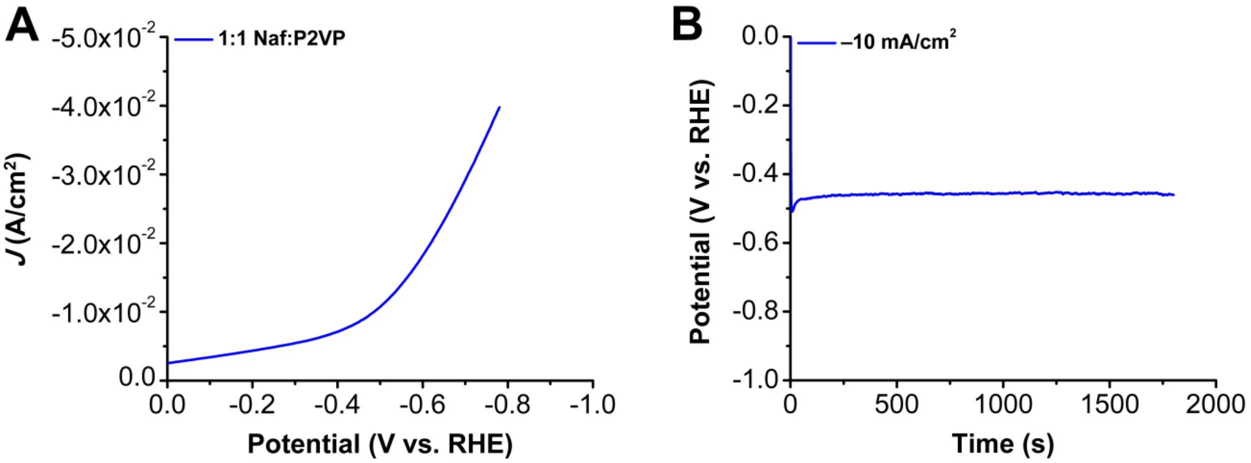

Figure S22. (A) LSV of 1 mM [Co] with 1:1 Naf:P2VP. (B) Stability test of 1 mM [Co] with 1:1 Naf:P2VP. Conditions: Ink Solution containing 1mM [Co], 1 mM pyridine, Vulcan Carbon, and 1:1 Naf:P2VP. 1 cm^2 coated carbon paper with ink solution working electrode, glassy carbon counter electrode, Hg/HgO reference electrode. Performed under N_2 in 1 M KOH. 10 mV/s scan rate.

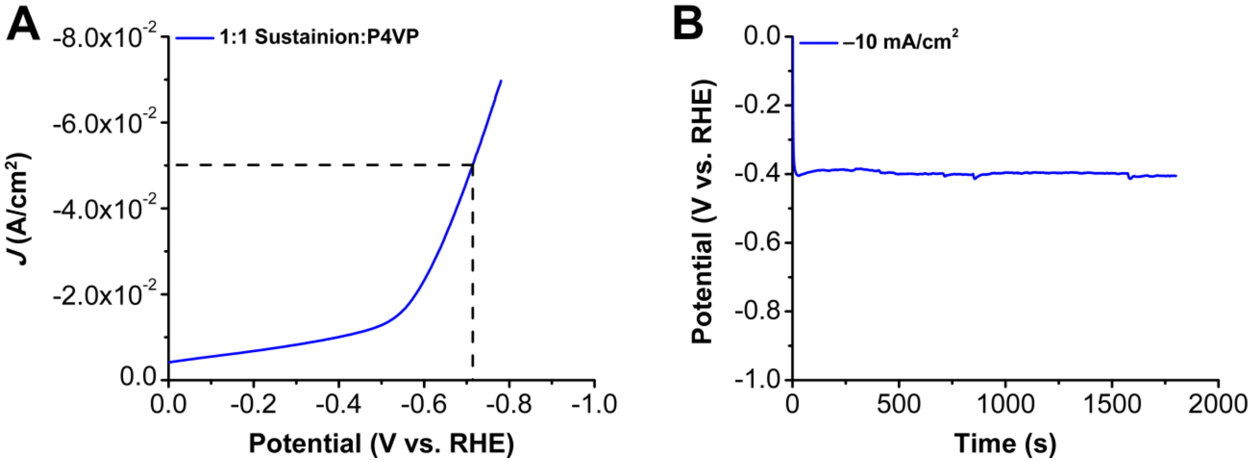

Figure S23. (A) LSV of 1 mM [Co] with 1:1 Sustainion:P4VP. (B) Stability test of 1 mM [Co] with 1:1 Sustainion:P4VP. Conditions: Ink Solution containing 1mM [Co], 1 mM pyridine, Vulcan Carbon, and 1:1 Sustainion:P4VP. 1 cm^2 coated carbon paper with ink solution working electrode, glassy carbon counter electrode, Hg/HgO reference electrode. Performed under N_2 in 1 M KOH. 10 mV/s scan rate.

Electron Microscopy

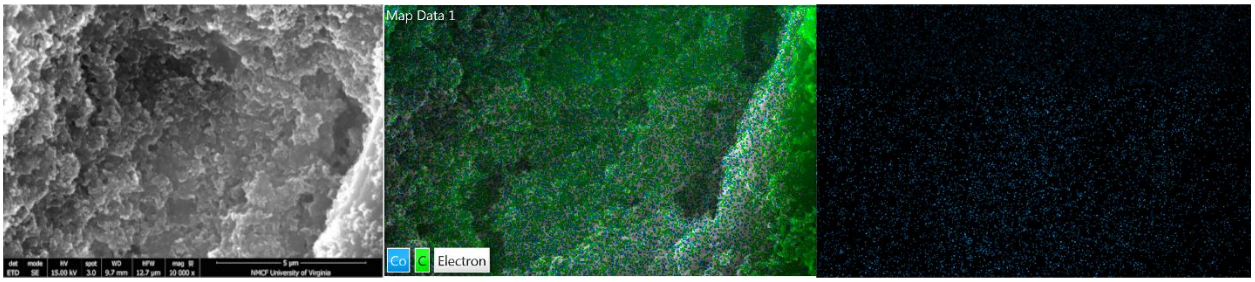

Figure S24. SEM-EDS images of [Co] with Nafion unused electrode.

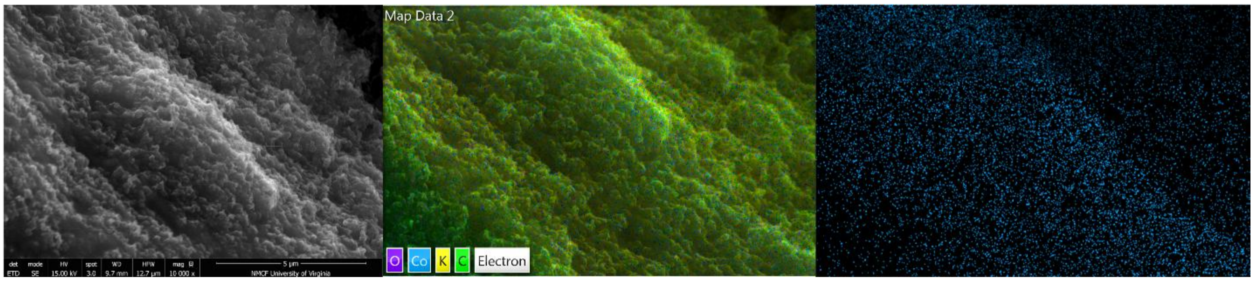

Figure S25. SEM-EDS images of [Co] with Nafion after LSVs.

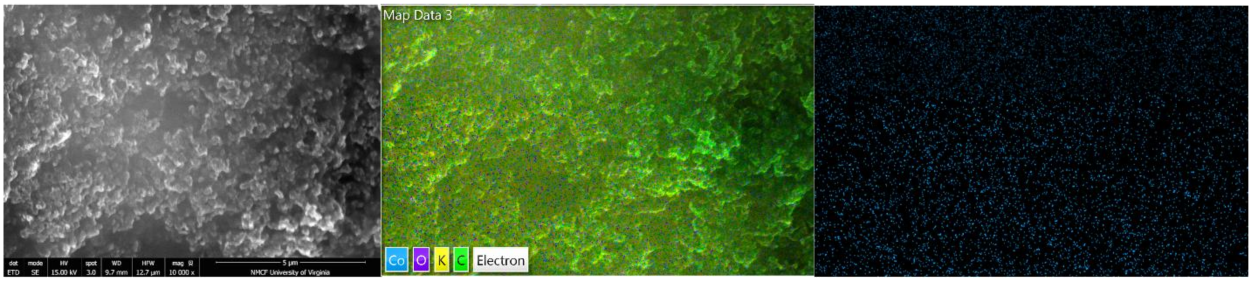

Figure S26. SEM-EDS images of [Co] with Nafion after –10 mA for 30 m chronopotentiometry experiment.

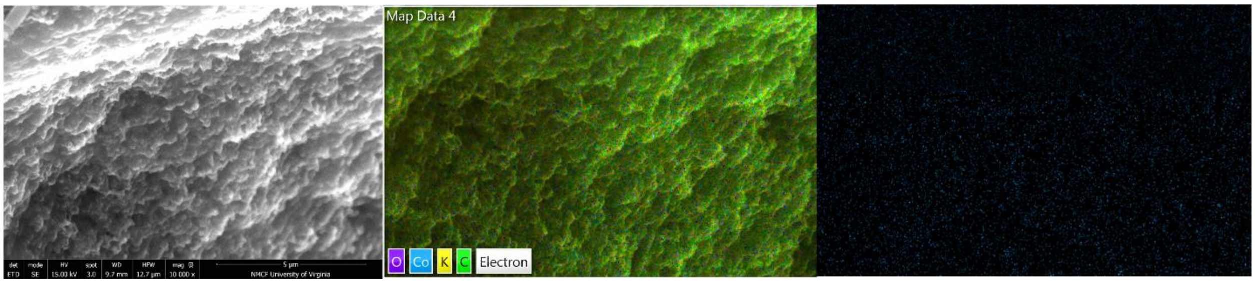

Figure S27. SEM-EDS images of [Co] with Nafion after –50 mA for 30 m chronopotentiometry experiment.

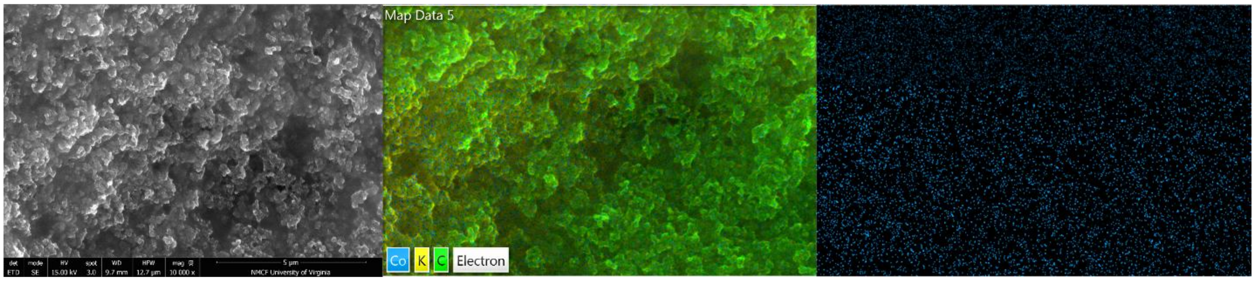

Figure S28. SEM-EDS images of [Co] with Nafion after –10 mA for 12 h chronopotentiometry experiment.

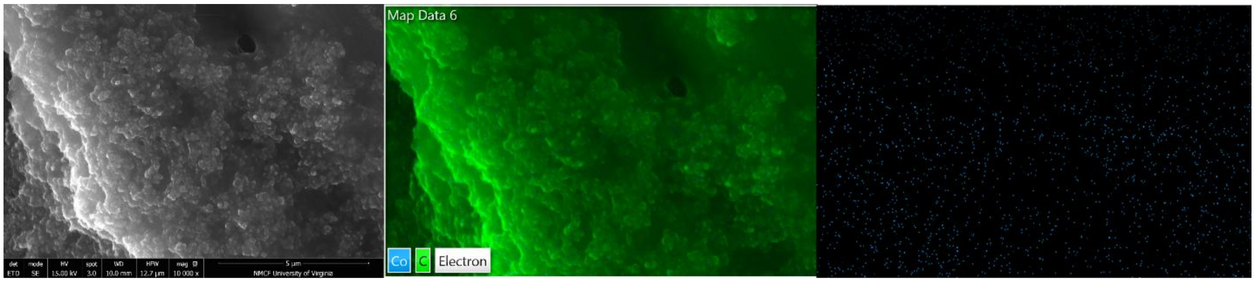

Figure S29. SEM-EDS images of [Co] with P4VP unused electrode.

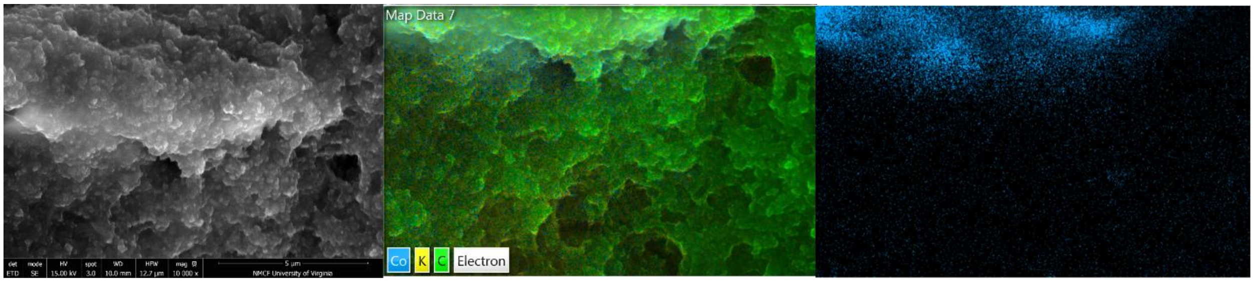

Figure S30. SEM-EDS images of [Co] with P4VP after LSVs.

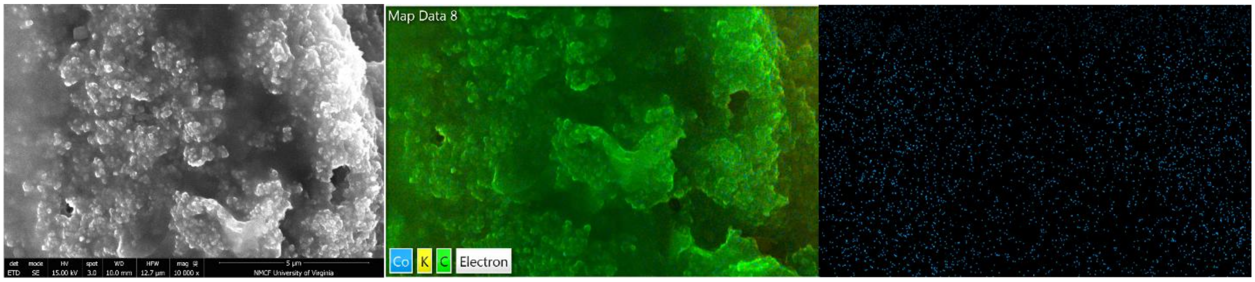

Figure S31. SEM-EDS images of [Co] with P4VP after –10 mA for 30 m chronopotentiometry experiment.

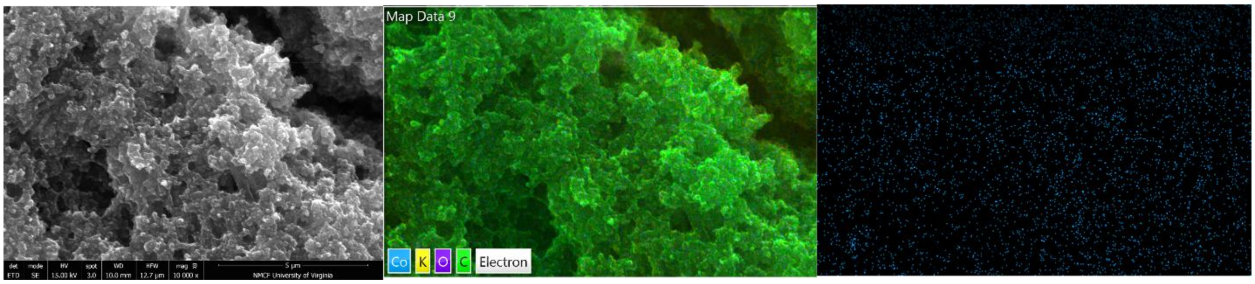

Figure S32. SEM-EDS images of [Co] with P4VP after –50 mA for 30 m chronopotentiometry experiment.

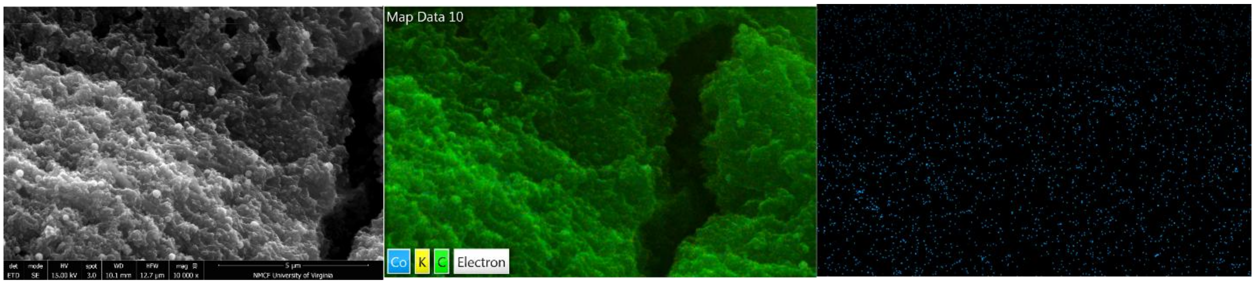

Figure S33. SEM-EDS images of [Co] with P4VP after –10 mA for 12 h chronopotentiometry experiment.

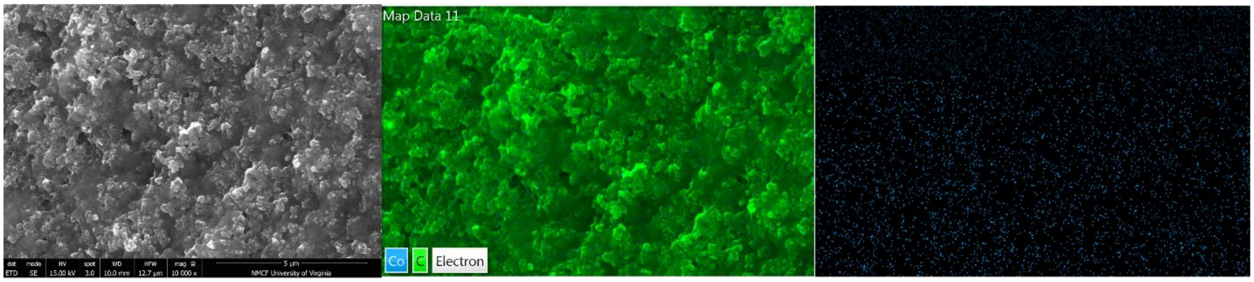

Figure S34. SEM-EDS images of [Co] with 1:1 Naf:P4VP unused electrode.

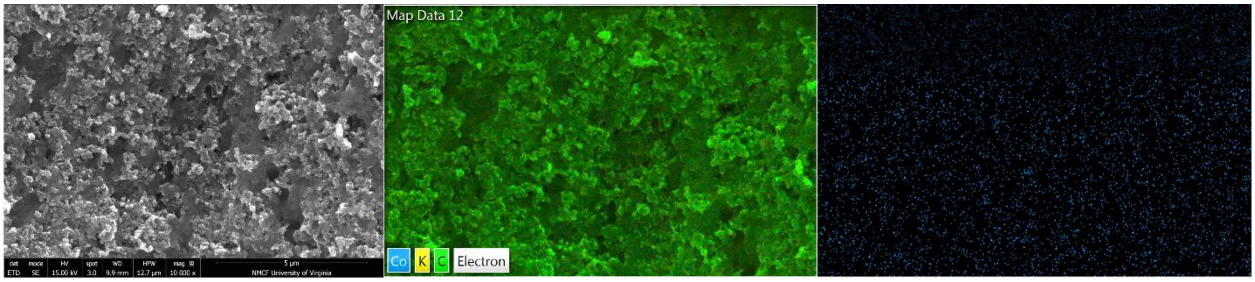

Figure S35. SEM-EDS images of [Co] with 1:1 Naf:P4VP after LSVs.

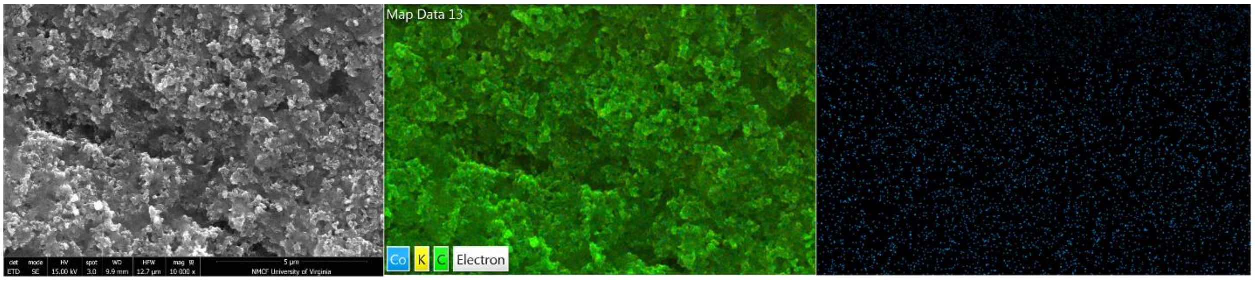

Figure S36. SEM-EDS images of [Co] with 1:1 Naf:P4VP after –10 mA for 30 m chronopotentiometry experiment.

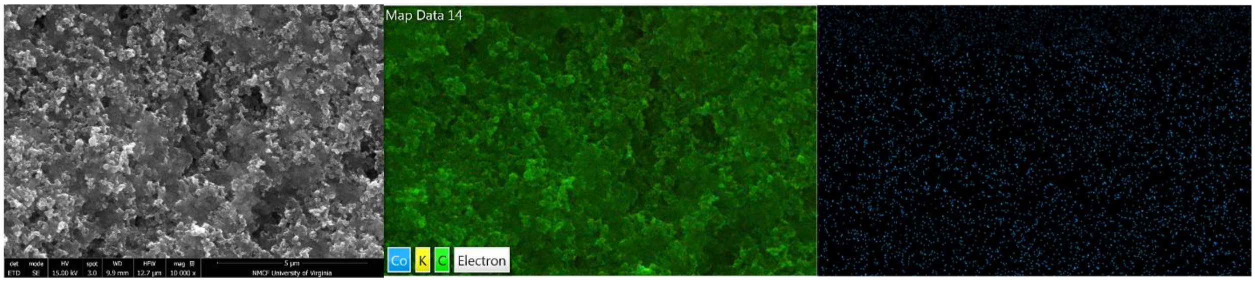

Figure S37. SEM-EDS images of [Co] with 1:1 Naf:P4VP after –50 mA for 30 m chronopotentiometry experiment.

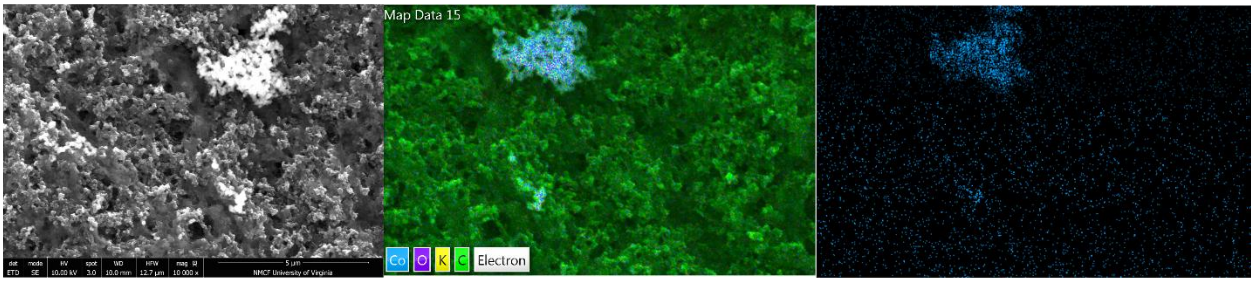

Figure S38. SEM-EDS images of [Co] with 1:1 Naf:P4VP after –10 mA for 12 h chronopotentiometry experiment.

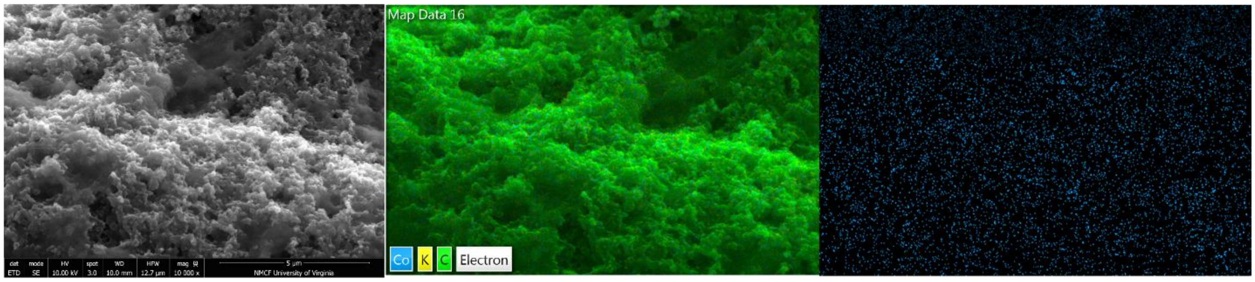

Figure S39. SEM-EDS images of [Co] with 1:1 Naf:P4VP after –50 mA for 12 h chronopotentiometry experiment.

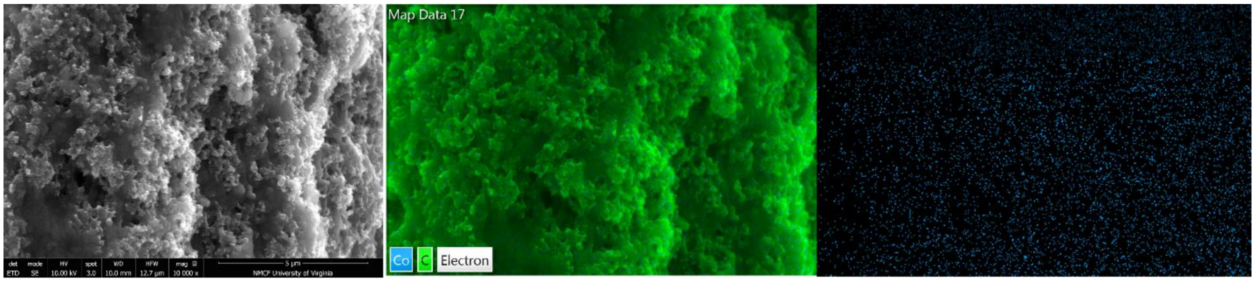

Figure S40. SEM-EDS images of [Co] with 1:3 Naf:P4VP unused electrode.

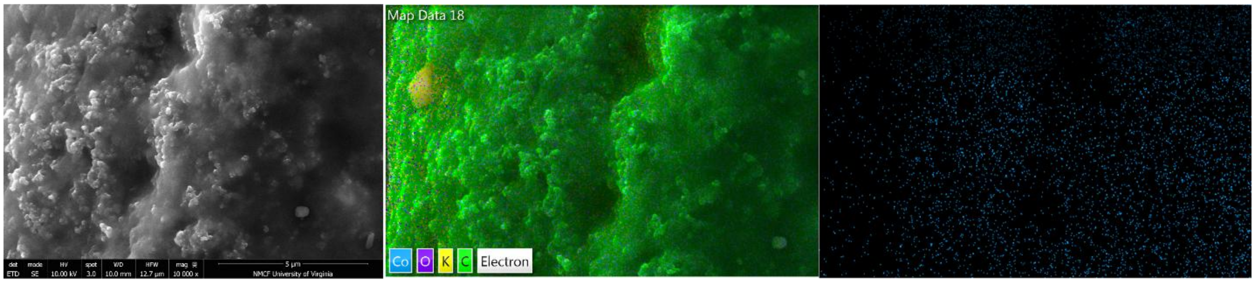

Figure S41. SEM-EDS images of [Co] with 1:3 Naf:P4VP after LSVs.

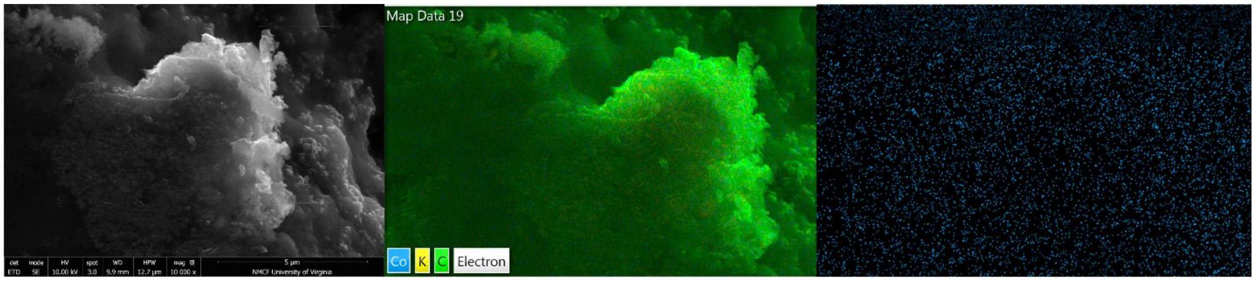

Figure S42. SEM-EDS images of [Co] with 1:3 Naf:P4VP after –10 mA for 30 m chronopotentiometry experiment.

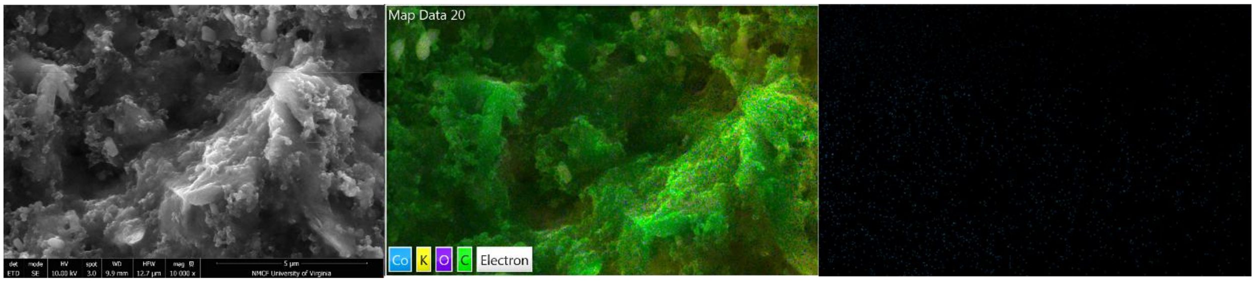

Figure S43. SEM-EDS images of [Co] with 1:3 Naf:P4VP after –50 mA for 30 m chronopotentiometry experiment.

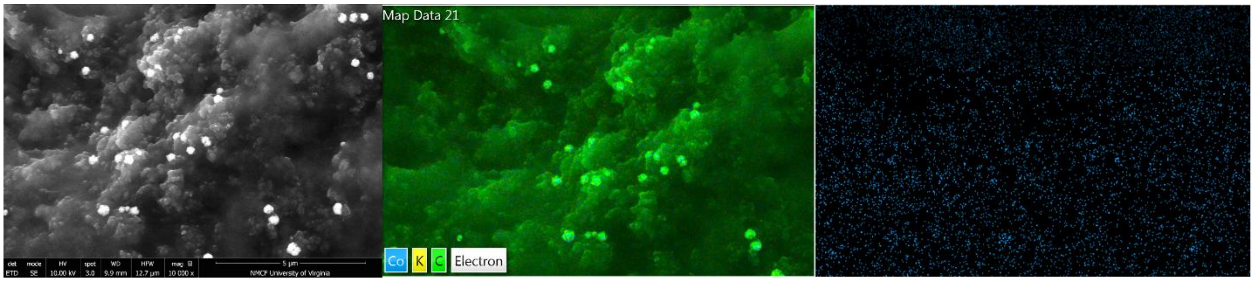

Figure S44. SEM-EDS images of [Co] with 1:3 Naf:P4VP after –10 mA for 12 h chronopotentiometry experiment.

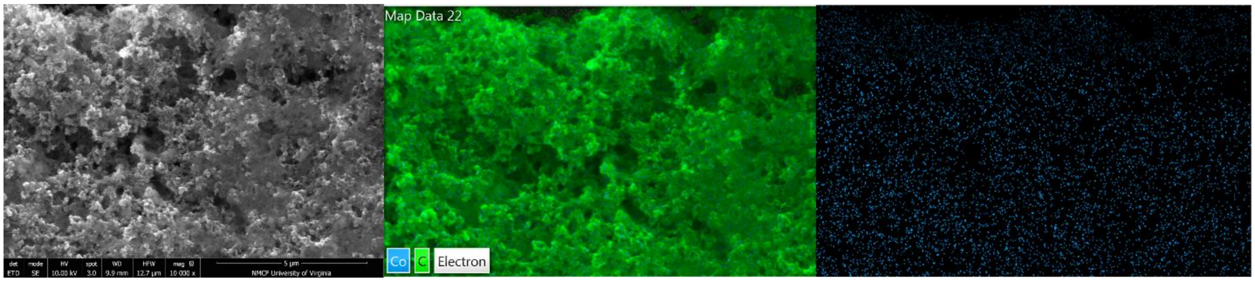

Figure S45. SEM-EDS images of [Co] with 3:1 Naf:P4VP unused electrode.

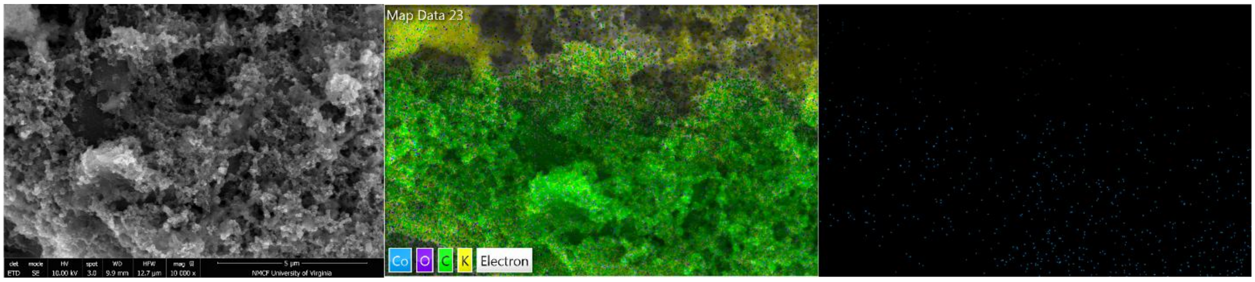

Figure S46. SEM-EDS images of [Co] with 3:1 Naf:P4VP after LSVs.

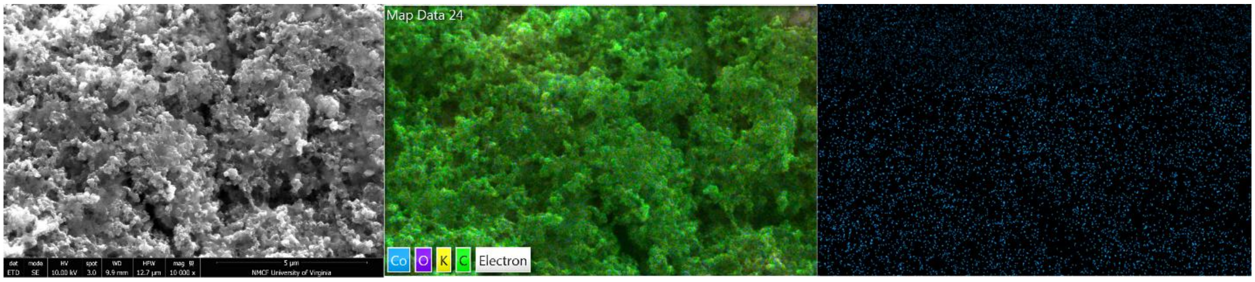

Figure S47. SEM-EDS images of [Co] with 3:1 Naf:P4VP after –10 mA for 30 m chronopotentiometry experiment.

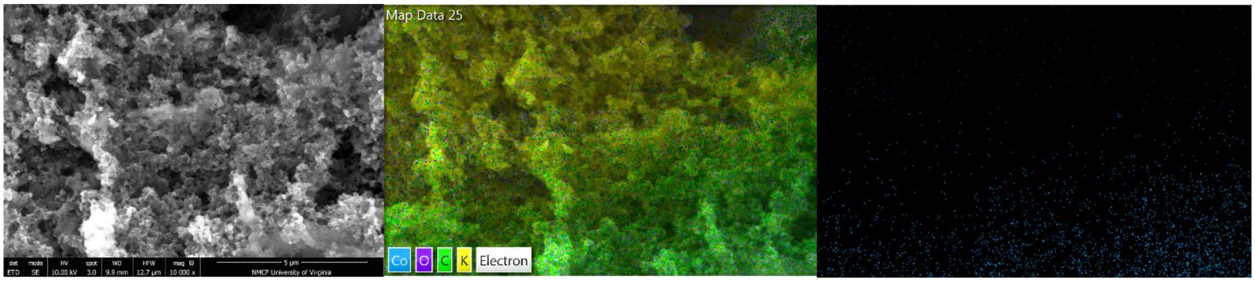

Figure S48. SEM-EDS images of [Co] with 3:1 Naf:P4VP after –50 mA for 30 m chronopotentiometry experiment.

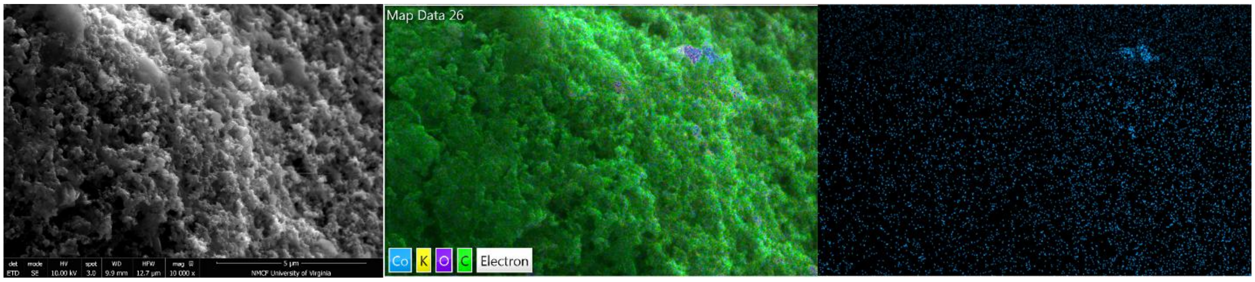

Figure S49. SEM-EDS images of [Co] with 3:1 Naf:P4VP after –10 mA for 12 h chronopotentiometry experiment.

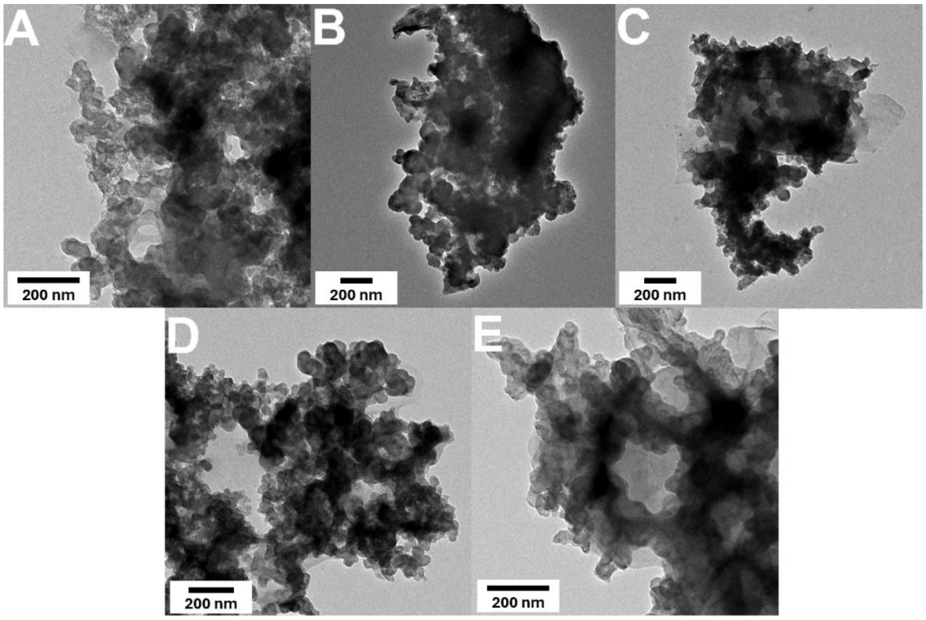

Figure S50. TEM images of Nafion electrodes (A) unused, (B) after LSVs, (C) after –10 mA for 30 m chronopotentiometry, (D) after –50 mA for 30 m chronopotentiometry, (E) after –10 mA for 12 h chronopotentiometry (replication of Figure 4A in main text for comparison).

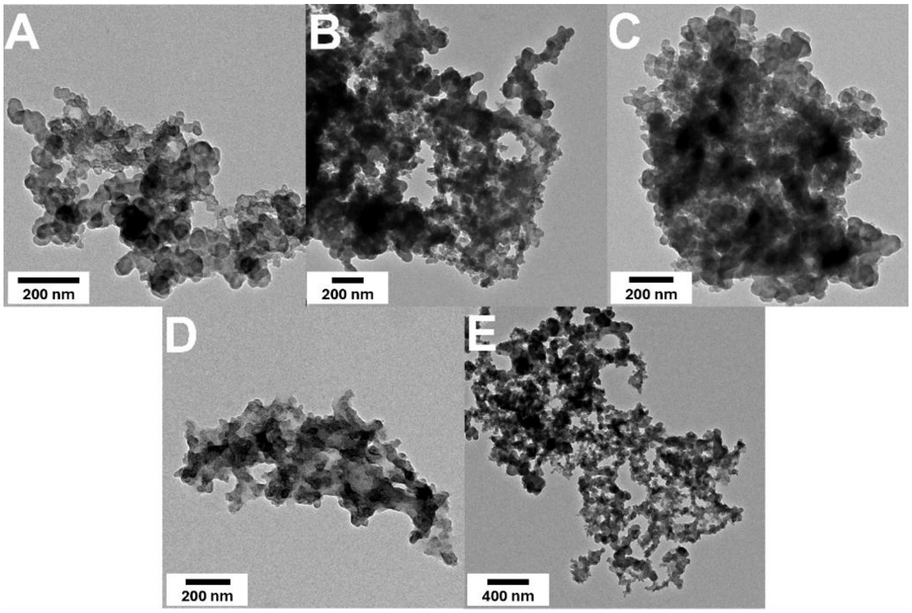

Figure S51. TEM images of P4VP electrodes (A) unused, (B) after LSVs, (C) after –10 mA for 30 m chronopotentiometry, (D) after –50 mA for 30 m chronopotentiometry, (E) after –10 mA for 12 h chronopotentiometry (replication of Figure 4B in main text for comparison).

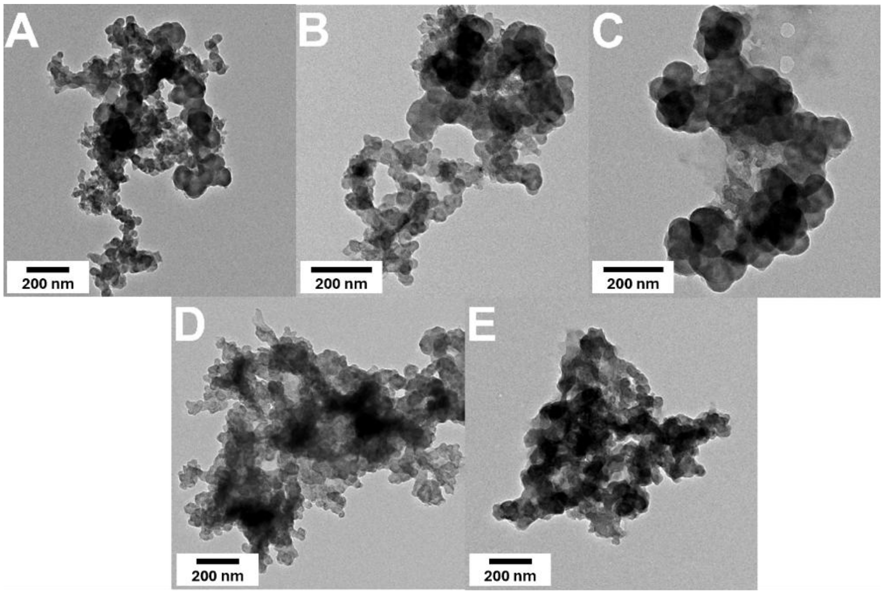

Figure S52. TEM images of 1:1 Naf:P4VP electrodes (A) unused, (B) after LSVs, (C) after –10 mA for 30 m chronopotentiometry, (D) after –50 mA for 30 m chronopotentiometry, (E) after –10 mA for 12 h chronopotentiometry.

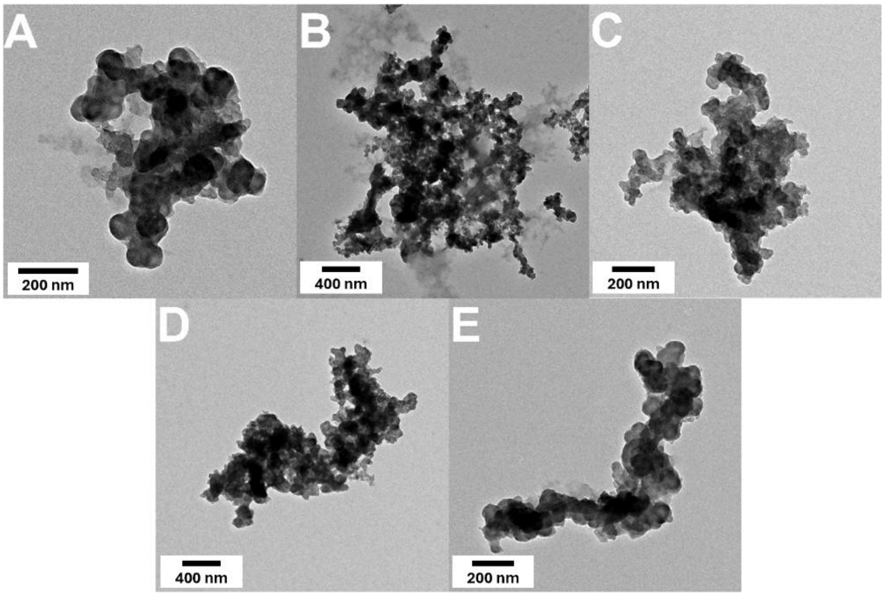

Figure S53. TEM images of 1:3 Naf:P4VP electrodes (A) unused, (B) after LSVs, (C) after –10 mA for 30 m chronopotentiometry, (D) after –50 mA for 30 m chronopotentiometry, (E) after –10 mA for 12 h chronopotentiometry.

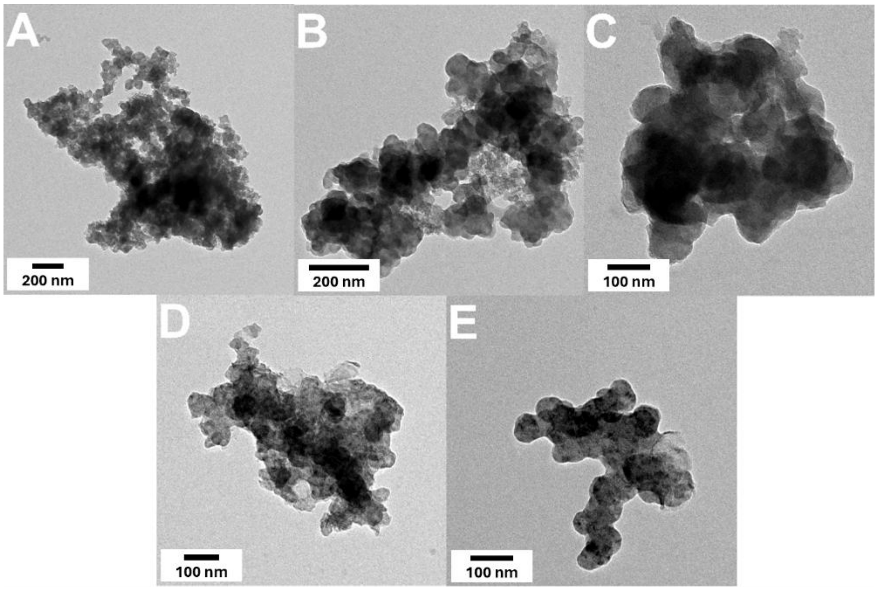

Figure S54. TEM images of 3:1 Naf:P4VP electrodes (A) unused, (B) after LSVs, (C) after –10 mA for 30 m chronopotentiometry, (D) after –50 mA for 30 m chronopotentiometry, (E) after –10 mA for 12 h chronopotentiometry.

UV-Vis Spectroscopy

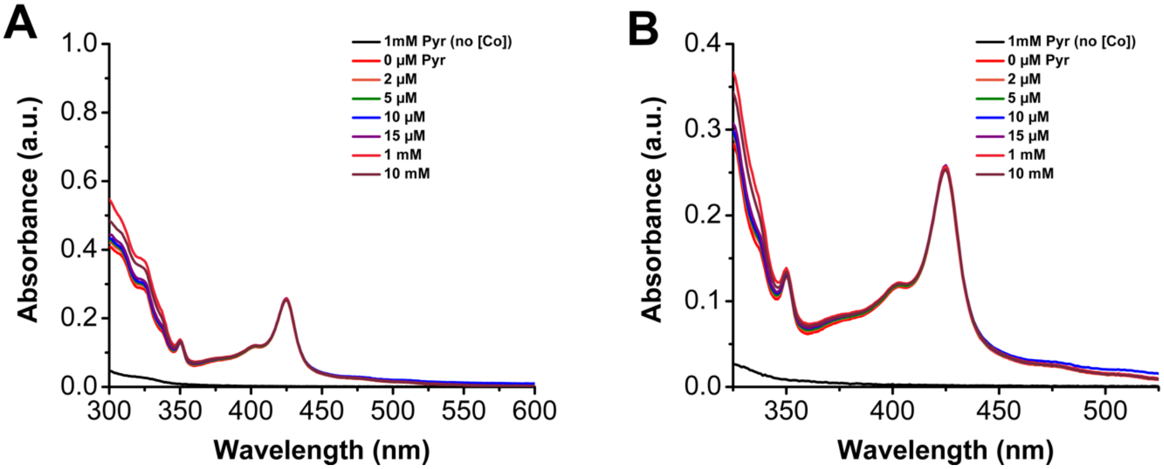

Figure S55. UV-Vis spectra of 10 μM [Co] with variable pyridine (Pyr) in DMF, (A) full window, (B) zoomed in to highlight absorbance band at 421 nm.

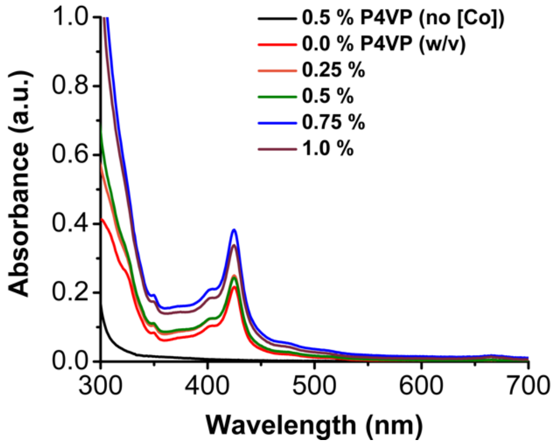

Figure S56. UV-Vis spectra of 10 μM [Co] with variable P4VP in DMF.

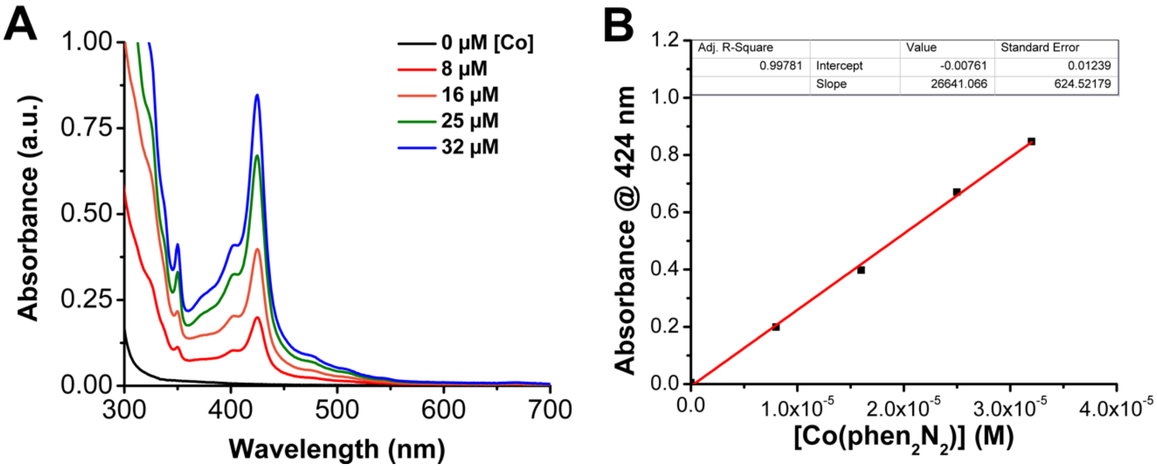

Figure S57. (A) UV-Vis spectrum of 0.5% w/v P4VP with variable [Co] in DMF. (B) Beer-Lambert plot of the absorbance band at 424 nm in (A).

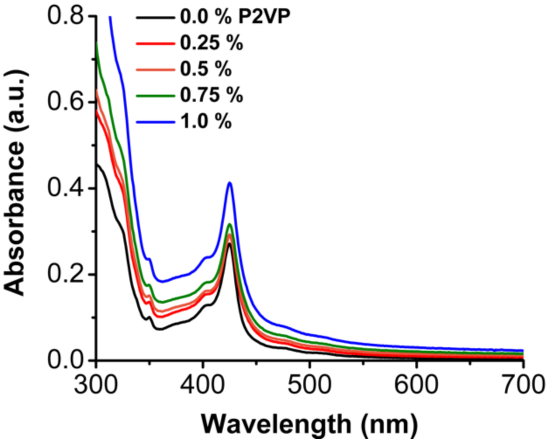

Figure S58. UV-Vis spectrum of 10 μM [Co] with variable P2VP in DMF.

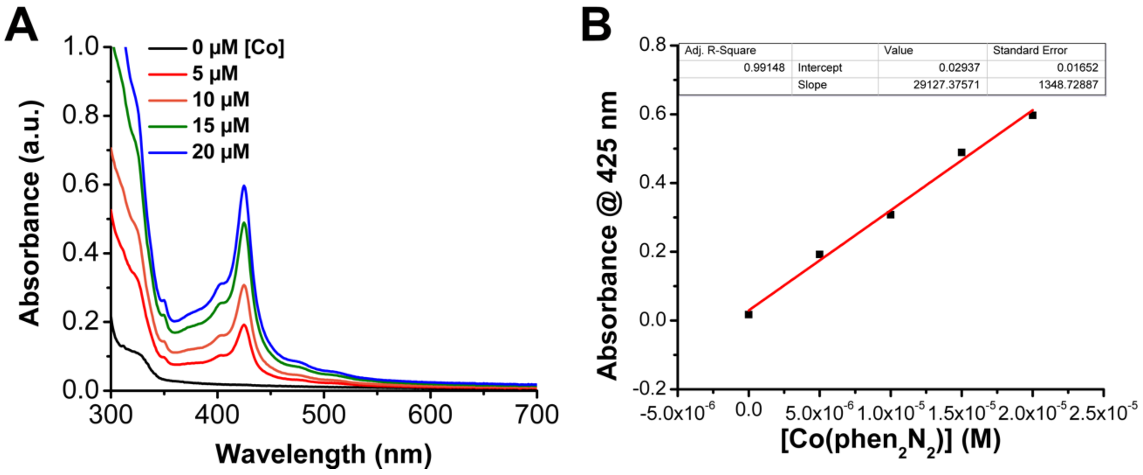

Figure S59. (A) UV-Vis spectrum of 0.5% w/v P2VP with variable [Co] in DMF, (B) Beer-Lambert plot of the absorbance band at 425 nm in (A).

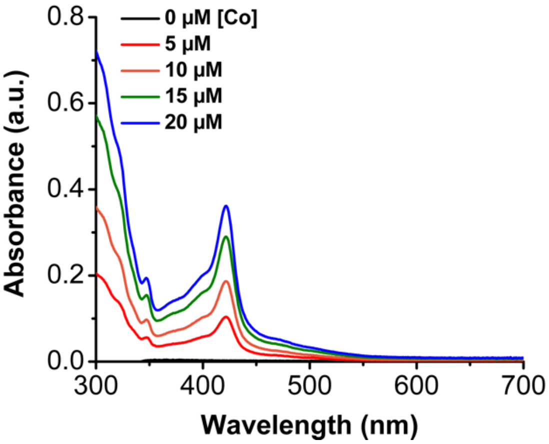

Figure S60. UV-Vis spectrum of variable [Co] in 90% DMF, 10% mixture of alcohols. Mixture of alcohols is composed of 39% 1-propanol, 39% IPA, 20% DI water, and 2% MeOH.

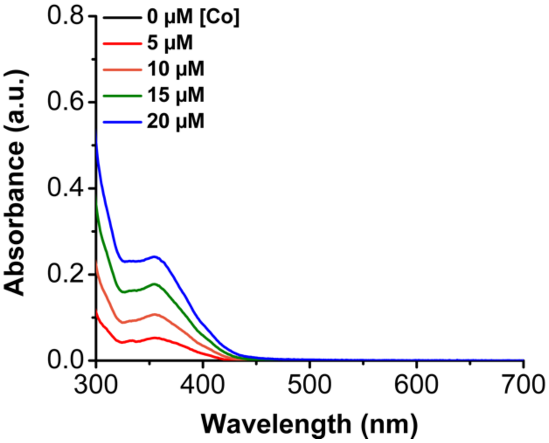

Figure S61. UV-Vis spectrum of variable [Co] in 90% DMF, 10% Nafion (0.5% w/v).

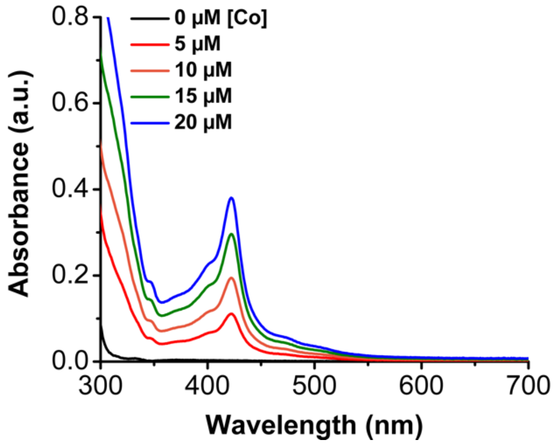

Figure S62. UV-Vis spectrum of variable [Co] in 90% DMF, 10% P4VP (5% w/v) in mixture of alcohols. Mixture of alcohols is composed of 39% 1-propanol, 39% IPA, 20% DI water, and 2% MeOH.

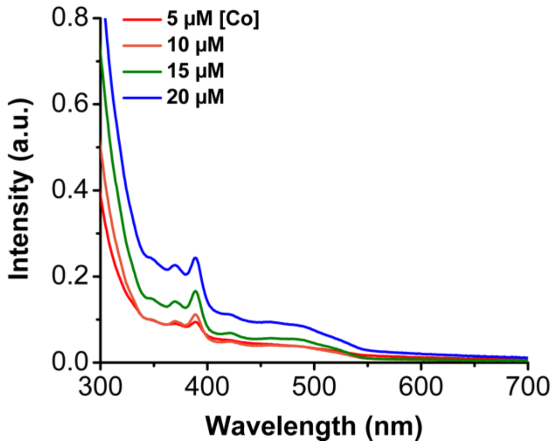

Figure S63. UV-Vis spectrum of variable [Co] in 90% DMF, 10% 1:1 Nafion:P4VP (5% w/v) in mixture of alcohols. Mixture of alcohols is composed of 39% 1-propanol, 39% IPA, 20% DI water, and 2% MeOH.

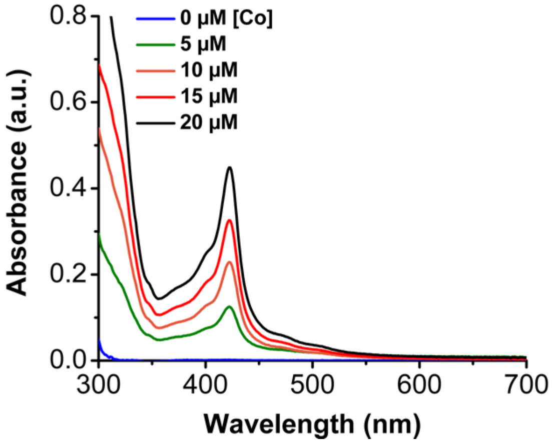

Figure S64. UV-Vis spectrum of variable [Co] in 90% DMF, 10% P2VP (5% w/v) in mixture of alcohols. Mixture of alcohols is composed of 39% 1-propanol, 39% IPA, 20% DI water, and 2% MeOH.

References

(1) Marshall-Roth, T.; Libretto, N. J.; Wrobel, A. T.; Anderton, K. J.; Pegis, M. L.; Ricke, N. D.; Voorhis, T. V.; Miller, J. T.; Surendranath, Y. A pyridinic Fe-N_4 macrocycle models the active sites in Fe/N-doped carbon electrocatalysts. *Nat. Commun.* **2020**, *11*. DOI: https://doi.org/10.1038/s41467-020-18969-6.

(2) Ogawa, S.; Yamaguchi, T.; Gotoh, N. Preparation of a Conjugated Tautomer of I ,I 4 :7,8-Diethenotetrapyrido-[2,1,6-de : 2',1',6'-gb : 2",1",6"-kl: Z"',l"',6"'-na] [I ,3,5,8,10,12] hexaazacyclotetradecine and its Metal Derivatives. *J. Chem. Soc., Perkin Trans. 1* **1974**, *1*, 976-978. DOI: https://doi.org/10.1039/P19740000976.

(3) Kawashima, K.; Márquez, R. A.; Son, Y. J.; Guo, C.; Vaidyula, R. R.; Smith, L. A.; Chukwuneke, C. E.; Mullins, C. B. Accurate Potentials of Hg/HgO Electrodes: Practical Parameters for Reporting Alkaline Water Electrolysis Overpotentials. *ACS Catal.* **2023**, *13* (3), 1893-1898. DOI: 10.1021/acscatal.2c05655.

(4) Niu, S.; Li, S.; Du, Y.; Han, X.; Xu, P. How to Reliably Report the Overpotential of an Electrocatalyst. *ACS Energy Lett.* **2020**, *5* (4), 1083-1087. DOI: 10.1021/acsenergylett.0c00321.

(5) Anantharaj, S.; Karthik, P. E.; Noda, S. The Significance of Properly Reporting Turnover Frequency in Electrocatalysis Research. *Angew. Chem. Int. Ed.* **2021**, *60* (43), 23051-23067. DOI: https://doi.org/10.1002/anie.202110352.
